# Wealth creation and disease burden: Evidence from Nigeria based on a Bayesian-VAR approach

**DOI:** 10.1371/journal.pone.0334709

**Published:** 2025-11-10

**Authors:** Milena Lopreite, Zhen Zhu

**Affiliations:** 1 Department of Economics, Statistics and Finance, “G.Anania” University of Calabria, Arcavacata of Rende, Italy,; 2 Kent Business School, University of Kent, United Kingdom; Obafemi Awolowo University, NIGERIA

## Abstract

Does wealth creation reduce disease burden for developing countries? In this paper a Bayesian-VAR (B-VAR) model is built to investigate the causal relationships between disease burden, wealth creation, life expectancy at birth and population growth over the period from 2006 to 2018 in Nigeria, a developing country with significant disease burden. Specifically, the use of the impulse response functions and the forecast variance decomposition functions reveal that wealth creation has the greatest impact on disease burden in Nigeria. Our results are consistent with different measurements of wealth creation, including trade in services, personal remittance received, and ease of doing business. We have also found a pronounced response of disease burden to life expectancy at birth and to population growth. Our results suggest that, in Nigeria, policies targeted at wealth creation, with a proper wealth redistribution, are strongly recommended to reduce disease burden and increase life expectancy at birth.

## 1. Introduction

The burden of disease represents for many developing countries a primary challenge, and a daily concern that strongly conditions the economic development and the well-being of the population. In these countries nearly two billion people have inadequate or no access to life-saving treatments and stay in poverty, facing wars and local conflicts [1].

The burden of disease prevents the socio-economic development by reducing productivity, life expectancy at birth, the level of education while slowing economic growth in general (Sachs and Malaney 2002, Cole and Neumayer 2006, Wang et al. 2020). The global impact of disease burden is a major concern not only for health reasons but also because disease burden affects either children or adults in the most part of their working life [2,3]. In 2019 the worldwide death rate for disease burden was nearly 7.5 percent with a higher incidence in the developing countries [4]. In the top ten global causes of death in 2019 there are neonatal conditions, malaria, tuberculosis, lower respiratory infections [5].

Beyond the mortality statistics the most used method to quantify the disease burden of a country is the approach that measures the global burden disease in terms of disability adjusted life years metric (DALYs). This variable was the first proposed measure of disease burden back in 1990 by the World Health Organization [6] and it has been updated in 2000–2004, and until 2010 thanks to the coordination of the WHO and the researchers of Harvard School of Public Health. Specifically, the DALYs is the combination of number of years of life lost (YLLs) through premature death, and the years lived with disability caused by the disease such as low back pain, iron-deficiency anemia, major depressive disorder, malaria, and schistosomiasis. This indicator quantifies both premature mortality (YLLs) and disability (YLDs, loss of health) within a population in terms of injuries and risk factors for the disease burden [6]. The disability adjusted life years (DALYs) together with the quality adjusted life years (QAYLs) give us information about the population’s health status, and they are both used to estimate the impact of the diseases in a country. However, while DALYs estimates the differences between the ideal case of living to old age in good health, and the current situation where healthy life is shortened by illness, injury, disability, and premature death, the QALYs is used to determine the best health care interventions for a good quality life [7–9].

Among the developing countries, Nigeria is one of the nations that still suffer from disease burden mostly due to malaria (17.1%), HIV/AIDS and tuberculosis (9.9%), diarrheal syndrome and neonatal disorders (9.9%), lower respiratory infection (8.3%) and neonatal encephalopathy due to birth asphyxia and trauma (5.8%) [10]. In terms of the number of years of life lost (YLLs) due to premature death, malaria remains the most important health issue to address in Nigeria with a strong increase of YLLs from 15.7% in the year 1990 to 23.2% in 2016 [10]. Thus, the findings above suggest malaria as a leading cause of YLLs and in turn of DALYs in Nigeria [10,11]. Analyzing the impact of this last indicator through the malaria incidence in Nigeria might be useful to get key insights relative to the “health gap” of this country for both an efficient resources allocation and health policy achievements [12]. In sum, the performance of Nigeria in terms of life expectancy at birth, YLLs, and YLDs lags that of other developing countries [10]. Specifically, according to the World Bank database in the year 2019, life expectancy at birth in the most developed countries was about 80 years, while in Latin America and Caribbean was 75 years, in South Asia was about 69 years which it goes down to 56 years in Africa, 61 years in sub-Saharan Africa and 55 in Nigeria.

In addition to the short life expectancy, the high fertility rate (5.3% in 2020 according to the [13] gives to Nigeria a youthful population with lower median age (18 years) than the rest of Africa (20 years) and the rest of the world (29 years) putting this country in a “demographic trap” that results in more poverty when people rely on having more children to provide them with economic security.

Finally, Nigeria often called “Giant of Africa” is the seventh most populous country in the world, with a population of about 206 million a population growth of 1.05% per year and more than 371 ethnic groups speaking over 500 languages [13]. According to the projection of OECD data Nigeria’s population is expected to reach 401 million by 2050, making it the third most populous country in the world.

The high disparities of Nigeria relative to different demographic, epidemiological and economic changes with respect to other developing countries make it impossible for the government to attain its development agenda as outlined in the Sustainable Development Goals by 2030 [14] that include eradicating poverty, combating malnutrition, expanding employment and educational enrolment. These aspects, combined with an inadequate health system characterized by inaccessibility to hospitals, poor hygiene, malnutrition, lack of access to safe drinking water, poor health infrastructure, insufficient financial investment, and lack of health personnel underline how the Nigeria urgently needs programs and interventions plans as well as a better regulation of the health care system.

A consequence of Nigeria’s “bleak development” is trapping the government in a state of “demographic fatigue” [15] in which for the lack of the financial resources the government becomes unable to deal with a range of threats such as crises, diseases etc.

Starting from these points, identifying the main leading variables of the disease burden and estimating their impact is strongly recommended to provide useful suggestions to policy makers and for having appropriate and accurate health development planning in Nigeria [9].

There are many studies, especially during the last decade, that analyzed which variables may affect the disease burden the most. For instance, according to a study of [16] over the period 1970–2000, a cardiovascular revolution may reduce the disease burden of a country because it led both to an increase of life expectancy at birth (+2 years) and higher education enrollment (of 3% points). Moreover, the Global Disease Burden (GBD) project for the year 2000 in line with the WHO programs revised several disability weights (i.e., mobility, self-care, pain and discomfort, cognition, interpersonal activities, vision, sleep and energy) providing a health evaluation with a description of the health condition in order to produce the best possible evidence-based description of health status, the causes of lost health, and likely future trends in health because they can impact on a disease burden of a country. Starting from this point [17] found for the AIDS an average disability weight of 0.5 followed by lower respiratory tract infection cases of 0.2 for both treated and untreated forms. Moreover, they showed that the mean disability weight for malaria cases was 0.20, for cancers at the terminal stage was 0.81 and infertility was 0.18. Finally, there are the asthma cases, with a mean disability respectively of 0.10 for the untreated cases and 0.06 for the treated ones.

Thus, the health of a population in terms of health conditions and healthcare (quality and accessibility) can be considered from an economic point of view, like a commodity, something that with proper investments can be of better quality reducing the spread of the diseases burden while improving well-being and economic performance. But reversely, does an improved economic performance reduce disease burden in developing countries?

The micro level evidence is mixed across developed countries and periods in the literature [18–20]. From a developing country’s perspective, “a good health status” is also directly influenced by income and in turn by wealth of a country: the demand for health requires resources and thus it grows if wealth increases. However, there exists a trade-off between health and growth: an expanding of the health sector may promote growth through better health conditions of the population, while a contraction of the healthcare sector could also free the resources necessary to promote growth [21]. In this context, [22] using an endogenous growth model investigated the relationship between health, wealth, and ageing population that can be relevant variables in explaining the disease burden of a country.

In the same way, [23] employing an endogenous growth model analyzed the relationship between income per capita and health (that he assumes analogous to a normal good): higher income leads to an increased demand for health, but the health status of a person also affects his or her income and earnings through different channels.

Finally, [23,24] in a growth model tried to examine the correlation between wealth of a country, health and longevity underlining the gain in longevity during the period of the 19th Century until the present in many countries and the deep divergence in the major causes of mortality between those periods defined “Epidemiological Transition”.

The analysis by the [10] on disease burden, investigating, as part of its policy recommendation, how the disease burden changes worldwide consequently to variations in population size and in the age structure. Specifically, the population growth increases the number of DALYs that in sub-Saharan Africa, raises respectively 47.6% caused by communicable, maternal, neonatal, and nutritional diseases, of 27.8% caused by non-contagious diseases, and of 32.6% caused by injuries. According to [17], population growth and aging are the key-drivers of the burden relative to non-infectious diseases.

More recently [25,26] found that economic development in the form of urbanization was positively correlated to non-communicable diseases such as diabetes in a large sample of 173 countries for the period from 1980 to 2008.

Starting from this context this work is guided by the following research questions: What are the main driving variables of the disease burden in Nigeria? Could an increase of wealth creation reduce the disease burden in Nigeria? Are there dynamic and significant relationships between disease burden, life expectancy at birth and population growth rate in Nigeria?

To address these questions, this paper contributes to the literature by adopting the following modeling strategy.

Firstly, different from previous studies, we consider the annual incidence of malaria as a disease burden proxy in Nigeria. This choice has two reasons: 1) According to DALYs there exists a link between disease and the population’s health: given that the health status is strongly and negatively influenced by a disease that, whether untreated, may degenerate and lead to demise –then, “a good status health” depends on the survival from a disease [27] Malaria is the leading cause of mortality in Nigeria: the 76 percent of the population in Nigeria live in high transmission areas for malaria while 24 percent of the population live in low transmission ones. The burden of malaria is three times greater among rural dwellers in comparison to urban dwellers: malaria and severe anemia were two times more prevalent in rural children than in their urban counterparts [28]. Nigeria had the highest number of global malaria cases (25 percent of global malaria cases) in 2018 and accounted for the highest number of deaths (24 percent of global malaria deaths) [11].

Secondly, we use the variable wealth instead of income, because it is an important indicator of financial stability, and it reflects the processes that lead to financial well-being and inequality. In fact, inequalities in wealth are in general accompanied by income inequalities. Moreover, an investigation on this measure, especially in low-income countries such as Nigeria, can offer valuable policy implications by analyzing individuals’ ability to turn income into wealth, as an indicator of need among the poorest households. Specifically, according to the literature we selected “Ease of Doing Business (EB)”, “Trade in Services (TIS)” and “Personal Remittance Received (PRR)” as proxies of the wealth creation process in developing countries such as Nigeria [29,30] to investigate the potential benefits a country may gain from sustained wealth accumulation. Moreover, we include these three measures for the following reasons: a) “Personal Remittance Received” (transfers and compensation of employees) was proven to be a significant source of income for families in the developing countries and to play a crucial role of co-insurance or risk mitigation in times of hardship more effective than other capital inflows such as foreign direct investment, public debt or official development assistance [30]; b) “Trade in Services” (the sum of service exports and imports divided by the value of GDP) is the ability of a country and its firms to compete in international markets. They play an increasingly important role in the global economy, in the growth and in the development of countries through the generation of opportunities for greater income, productivity, employment, investment and trade [31,32]; c) “Ease of Doing Business” index (it illustrates the distance of an economy to the “frontier”, which represents the best performance observed on each “Doing Business” topic) and its ranking is important for two reasons: First, foreign direct investments (FDI) will lead to growth. Second, there will be trickle-down impact of growth that will lead to poverty alleviation [33]. In this way we can better capture the “wealth effects” on disease burden with respect to consider only a single variable. Since our dataset covers a limited period due to data availability, we analysed the effects of these variables on disease burden by inserting them into the model one at a time. This approach allowed us to search for consistent results across all three cases.

Thirdly, in modeling disease burden dynamics, population growth and life expectancy at birth are inherently interconnected with health outcomes and economic conditions, making them critical endogenous variables for our analysis. Population growth directly influences the scale and structure of disease burden. Rapid population increases can strain healthcare infrastructure and resources, exacerbating the prevalence and impact of communicable and non-communicable diseases [34]. Moreover, demographic changes shape labor markets and economic productivity, thus intertwining with wealth creation and public health indicators [34]. In Nigeria, a country with one of the highest population growth rates globally, fluctuations in population size can significantly affect disease dynamics and resource allocation (OECD Data 2021). On the other hand, life expectancy at birth serves as a comprehensive summary measure of population health and reflects cumulative impacts of disease burden, healthcare quality, and socioeconomic conditions [35]. It is also a key indicator linking health outcomes to economic development, as healthier populations tend to support more robust wealth creation processes [34].

Therefore, we propose a novel and original study on disease burden in Nigeria investigating the possible interplay between disease burden, wealth creation, life expectancy at birth and population growth. Given the small size of our sample due to data availability, we use a Bayesian-VAR (B-VAR) model based on Minnesota prior that shows an efficient performance with respect to VAR approach and other B-VAR specifications.

The reminder of this paper is organized as follows. Section 2 introduces the B-VAR methodology. Section 3 describes the data and shows the results of impulse response functions and variance decomposition functions. Section 4 discusses these results. Section 5 evaluates the fit of the B-VAR model- based on Minnesota prior- by comparing its forecasting performance with the VAR model and the B-VAR model based on Normal Wishart prior, using the Root Mean Square Error (RMSE) and Mean Absolute Error (MAE) indicators.. Section 6 concludes with policy implications.

## 2. Methodology

Vector Autoregression (VAR) models have been standard tools in macroeconomic analysis and forecasting for decades [36,37]. Their popularity stems from their simple formulation and their success in capturing dynamic linear relationships between time series without imposing the restrictions found in structural VAR models.

However, VAR models encounter several challenges when they are estimated using a frequentist approach:

- **Overfitting Risk**: A traditional VAR model performs best with a small number of variables. Using too many variables, or even a moderate number, can lead to “overfitting” because the number of unrestricted parameters that can be reliably estimated is limited.
- **Omitted Variables Bias**: Since VAR models usually include only a few variables, they can be poor for forecasting and structural analysis due to omitted variables bias [37,38].
- **Loss of Degrees of Freedom**: Increasing the lag length in a VAR model leads to a loss of degrees of freedom. This results in large standard errors and imprecise point estimates, weakening the reliability of the findings.

These problems are particularly relevant in our context, as data limitations restrict our analysis to a short period (2006–2018).

The Bayesian VAR (B-VAR) approach offers a robust solution to the overfitting and poor forecasting performance often seen with VAR models, especially when dealing with short datasets, weak sample information, or a large number of parameters [36,39–42].

B-VAR model leverages Bayesian inference through parameter shrinkage, which significantly improves both dynamic analysis and forecasting accuracy. This method works by:

- **Reducing Estimation Error**: It helps reduce the estimation error and introduces only a small bias in the parameters. This is achieved by imposing restrictions (*priors*) on the coefficients, effectively assuming that coefficients of shorter lags are more likely to be close to zero [39,40].
- **Integrating Prior Knowledge**: The core strength of the Bayesian VAR lies in using priors. This allows researchers to combine the information from the sample data with their existing knowledge or beliefs about the coefficients to derive a more informed posterior distribution. This means the model achieves better results without needing highly restricted models (i.e., models with many coefficients set to zero).

Finally, the choice of prior is a critical step in Bayesian inference. If a prior is too “loose” (not restrictive enough), it becomes difficult to prevent overfitting. Conversely, if it is too “tight” (overly restrictive), it limits the data’s ability to influence the estimation, preventing the model from fully capturing the underlying signal. Researchers carefully set priors based on available information regarding the parameters’ nature or use informative priors to reflect their expert beliefs.

The careful specification of priors helps solve two fundamental problems in model estimation:

- Equations with too many free parameters tend to pick up excessive noise.
- Equations with too few parameters fail to capture the true signal.

Ultimately, the use of priors in B-VAR provides a flexible solution, enabling a balanced trade-off between overfitting data and enhancing signal extraction capabilities.

In this paper we analyze the dynamic relationships between disease burden, wealth, life expectancy at birth and population growth in Nigeria without imposing *a priori* economic restrictions or causality directions. Given the limited size of our sample due to data availability, we cannot apply the VAR models as we may occur in problems of over-parametrization (estimating too many variables with relatively little data) [39–45]

Employing Bayesian techniques over the period from 2006 to 2018 we overcome this problem by accumulating evidence from data to determine the chances of realizing certain parameter values. In this way we incorporate the researcher’s belief using specified priors that transform the VAR model into a more parsimonious model (Bayesian VAR model) respectively reducing the VAR dimensionality and considering the researcher’s uncertainty beliefs.

While in classical inference (or frequentist approach) the data generating process (DGP) are distributed according to the properties of a random variable (i.e., they are stochastic) and the parameters are known and fixed, the idea behind the Bayesian approach is that data are given and the parameter vector is unknown and it contains a number of random variables with an assigned probability distribution (*prior probabilities*).

In this case the prior probability distribution reflects the researcher’s subjective beliefs about the value of the parameter vector chosen before the data inspection (non-sample information), while the likelihood function captures the information in the sample.

Given the data vector Y and the parameter of interest θ by applying the Bayes theorem the prior information p(θ) is formally combined with the information contained in the data as captured by the likelihood function $L\left(\frac{Y}{\theta }\right)$ to form a posterior distribution $p\left(\frac{\theta }{Y}\right)$ that generally has the same density function of the prior:


$$p\left(\frac{\theta }{Y}\right)\propto L\left(\frac{Y}{\theta }\right)*p(\theta )$$ (1)

Where $p(\frac{\theta }{Y}\backslash )$ contains all the information that we have on the parameter vector θ (prior beliefs) after having updated our prior views by looking on the data (likelihood function). Moreover, ∝ shows that in Equation (1) the right-hand side and the left-hand side are proportional.

To illustrate the usage of the Bayesian analysis we consider the reduced form of a VAR (q) model:


$$\begin{array}{c} Y_{t}=\sum {\mathrm{\backslash nolimits}}_{j=\text{1}}^{q}BY_{t-j}+u_{t}\text{t}=\text{1},\text{2}\ldots ,\text{T}u_{t}\sim N(\text{0},\sum {\mathrm{\backslash nolimits}}_{u}) \end{array}$$ (2)

Where $Y_{t}$ is an N×1 vector of endogenous variables in period t, B is an N×N matrix of coefficients corresponding to the j-th lag of $Y_{t}$ and, $u_{t}$ is a set of error terms with mean zero, zero autocorrelation and variance-covariance matrix $\sum_{u}$.

The reduced form summarizes the sample information in the data where $u_{t}$ is serially uncorrelated and orthogonal to the regressors in each equation.

In this study we impose on a VAR(q) model a conventional Minnesota-Litterman prior [39] that shrinks the VAR estimation towards a multivariate random walk model. The beliefs of the investigator can be specified in the following way:


$$\text{B}\sim \text{N}(\beta_{\text{0}},V)$$ (3)

In which $\beta_{\text{0}}$ is the prior mean and V is chosen to “center” the individual equations around a random walk with drift.

By applying the random walk hypothesis to Equation (2) we obtain that the diagonal elements of $B_{\text{1}}$ (coefficient matrix on the first lag) shrink toward one while the remaining coefficients of $B_{\text{1}}$,…..$B_{p}$ (prior mean $\beta_{\text{0}}$ outside the diagonal) shrink toward zero.

Specifically, the goal of the Minnesota parsimonious specification is to consider a normal prior on a set of parameters with the matrix of variance-covariance $\sum_{u}$ known, fixed and selected diagonal.

If we replace $\sum_{u}$ with its estimation from the data, $V_{\text{0}}$ we obtain the prior distribution of *B* based on the Minnesota prior as a *priori* normal and conditional upon the variance-covariance matrix $\sum_{u}$:


$$\begin{array}{c} p(B/\sum {\mathrm{\backslash nolimits}}_{u})\sim N(\beta_{\text{0}}{,V}_{\text{0}}) \end{array}$$ (4)

Following [46] since the prior covariance matrix $V_{\text{0}}$ is diagonal we do not specify all the elements of $V_{\text{0}}$ but we select only the coefficients in Equation (1) and $V_{ijj}$ will be the $V_{\text{0}}$ diagonal elements. The implementation of the Minnesota prior lead to set:


$$V_{ijj}=\left\{ \begin{array}{c} \frac{\lambda_{\text{1}}}{q^{\text{2}}}\text{for coefficients on own lags}\\ \frac{\lambda_{\text{2}}}{q^{\text{2}}}*\frac{\sigma_{ii}}{\sigma_{jj}}\text{for coefficients on lags of variable}j\neq i\\ \lambda_{\text{3}}*\sigma_{ii}\text{for coefficients on exogenous variables}\\ \lambda_{\text{4}forlagdecay} \end{array}\right.$$ (5)

Where q is the order of the B-VAR model, $\sigma_{ii}$ is the ii-^th^ element of the $V_{\text{0}}$ and $\sigma_{jj}$ is the jj-^th^ element of the $V_{\text{0}}$.

In specifying the Minnesota prior the researchers set up the key-hyperparameters λ that are related to the shrinkage of the parameters and to the control the overall prior variance of all VAR coefficients as follows:

- $\lambda_{\text{1}}$ weighs the relative importance of prior and data. If $\lambda_{\text{1}}$ is large the likelihood would be relatively flat (uninformative) and the posterior distribution mirrors the sample information. Otherwise, if it is very small and shrinks to zero the prior outweighs any information in the data improving the dynamical analysis and the forecasting performance of the reduced VAR model. In this last case, the posterior approaches the prior.
- $\lambda_{\text{2}}$ controls the cross-variables weight. If $\lambda_{\text{2}}$ is close to zero, the elements outside the diagonal of matrix B shrink towards zero. Otherwise, there is no difference between the lags of the dependent variable and the lags of the other variables.
- $\lambda_{\text{3}}$ sets the information associated with the exogenous variables.
- $\lambda_{\text{4}}$ captures the lag decay accounting for the varying scale and variability within the data. When the $\lambda_{\text{4}}$ is set to 1, it implies a linear decay. However, if it is set to a value greater than zero, two functional forms for decay become possible: harmonic or geometric decay.

After setting the prior covariance, since the Minnesota (Litterman) prior has a normal distribution, the B-VAR approach yields a normal distribution of the posterior too. The **Minnesota prior** and the **Normal-Wishart prior** were selected for this analysis due to their established effectiveness in Bayesian Vector Autoregression (BVAR) models. The *Minnesota prior*, known for its parsimony, addresses the issue of over-parameterization by shrinking the coefficients of lagged variables toward a random walk specification. This reduces the number of parameters to be estimated, leading to more stable and reliable results, particularly with shorter time series (our study). The *Normal-Wishart prior* is a conjugate prior for the parameters of a multivariate normal model, offering a computationally efficient and analytically tractable framework. Together, these priors allow to capture both complex interdependencies in the data while preventing overfitting-a key advantage over other, less constrained priors.

## 3. Data

To analyze the disease burden in Nigeria we consider a B-VAR model including the annual data of malaria incidence as a proxy of disease burden (BD) and as healthcare indicator along with life expectancy at birth (LFE), and population growth (POP) as demographic and health indicator. Finally, Ease of Doing Business index (EB), Trade in services (TIS) and Personal Remittance Received (PRR) are used as economic and wealth indicators. We consider the malaria incidence as a proxy of disease burden because it was responsible for 60 percent of outpatient appointments and 30 percent of admissions [28]. Moreover in 2019 with 229 million cases of malaria and 409,000 deaths Nigeria was home to 94 percent of all malaria cases and deaths [11]. Consequently, in Nigeria, malaria is seen both as a disease of poverty and a cause of poverty.

In addition, we include three different measures of “wealth effects” in Nigeria, “Trade in Services” (TIS), “Personal Remittance Received” (PRR), and “Ease of Doing Business” index (EB), which were drawn from the World Bank database.

**Fig 1:**
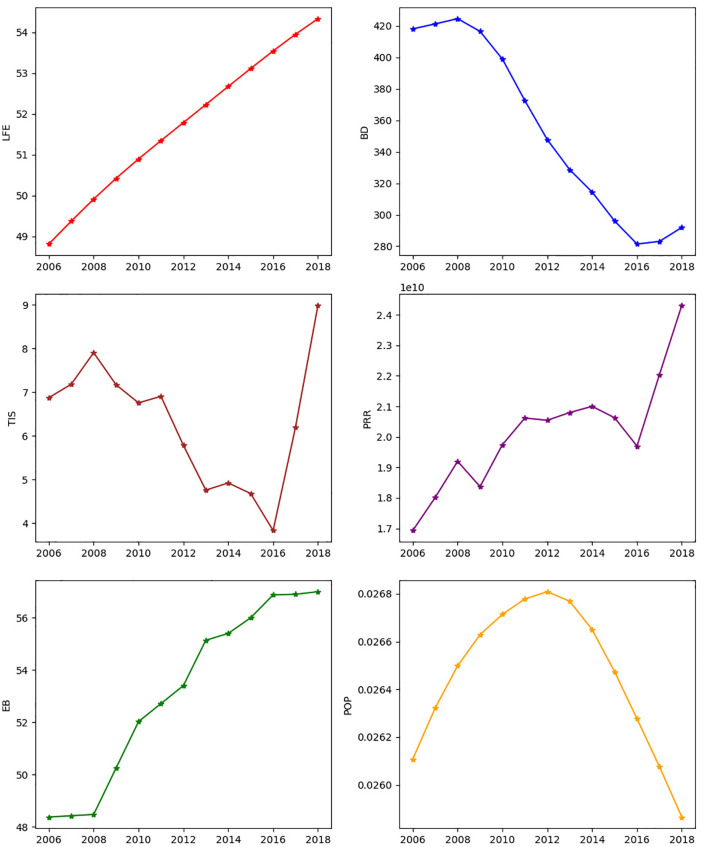
BVAR variables time series.

The Fig 1 graphically illustrates the time series trends for six key variables in Nigeria from 2006 to 2018. These time series offer crucial preliminary insights into the trajectories of each variable and hint at potential interdependencies.

Life expectancy at birth (LFE), depicted in the top-left panel, demonstrates a consistent and encouraging upward trend throughout the observed period, rising from just under 49 years in 2006 to approximately 54 years by 2018. This steady increase suggests a continuous improvement in overall health outcomes and living standards in Nigeria, potentially reflecting advancements in healthcare access, sanitation, and nutrition.

In contrast, the disease burden (BD), measured by annual malaria incidences and shown in the top-right panel, exhibits a significant decline from around 420 cases in 2006 to approximately 280 cases by 2016, before showing a slight rebound in 2017 and 2018. This pronounced reduction until 2016 strongly indicates successful public health interventions and control measures against malaria during that period. The subsequent slight increase warrants further investigation, as it could signal a slowdown in progress or new challenges in disease control.

The population growth rate (POP), presented in the bottom-right panel, follows a distinct bell-shaped curve. It gradually increases from around 0.0261 in 2006, peaking around 0.0268 in 2012, and then steadily declines to approximately 0.0261 again by 2018. This pattern suggests a period of accelerating growth followed by deceleration, which could be influenced by various demographic factors such as birth rates, mortality rates, and migration, or even by the initial successes in health improvements impacting infants and child mortality.

Among the wealth creation indicators, Trade in Services (TIS), shown in the middle-left panel, exhibits notable fluctuations throughout the period, remaining relatively stable with minor dips until a sharp increase from 2016. This late-period surge suggests a recent expansion in Nigeria’s service sector or increased participation in international trade of services. This could be indicative of policy shifts, increased foreign investment in service industries, or a growing domestic service economy.

Personal Remittances Received (PRR), in the middle-right panel, shows a general upward trend, albeit with some minor fluctuations, increasing from approximately 1.7 in 2006 to around 2.4 by 2018. This consistent growth points to growing financial inflows from abroad, highlighting the increasing contribution of the Nigerian diaspora to the country’s economy. These remittances can significantly impact household incomes, consumption, and investment, thereby influencing overall wealth creation.

Finally, the Ease of Doing Business (EB) index, displayed in the bottom-left panel, demonstrates a consistent and steady improvement from just under 49 in 2006 to nearly 57 by 2018. This continuous upward trajectory suggests an increasingly favorable business environment in Nigeria, likely a result of ongoing government reforms aimed at reducing bureaucratic hurdles, improving regulatory frameworks, and fostering investment. Such improvements are critical for attracting both domestic and foreign investment, stimulating economic activity, and ultimately contributing to wealth creation.

Collectively, the data suggest that from 2006 to 2018, Nigeria experienced positive developments across several key domains. There were clear improvements in life expectancy and economic indicators, coupled with a significant reduction in disease burden. This broad pattern points to potential positive interactions between health outcomes and economic growth, where healthier populations may be more productive, and economic prosperity can fund better healthcare and living conditions. The trends, such as the initial decline and later stabilization/slight increase in disease burden, the bell-shaped curve of population growth, and the late surge in trade in services, underscore the dynamic and complex nature of these interrelationships. These preliminary observations from Fig 1 provide the basis for employing the Bayesian VAR model, to investigate the causal dynamics and the relationships between these key variables when the sample is short and we don’t impose theoretical a-priori restrictions.

### 3.1 Results

In order to investigate the dynamic relationships between our variables, we set the B-VAR model of order two according to the Bayesian criterion and we impose a conventional Minnesota prior setting to the hyperparameters as follows: a) λ1 (prior information) is set to a low value since the prior beliefs are informative; b) λ2 (cross variables lag) is assumed to be greater than zero since the information of cross variable lags is important; c) λ3 (exogenous variables) is set close to zero since the exogenous variables are not the focus of our study; d) λ4 (lag decay) is fixed and equal to 1.

**Fig 2:**
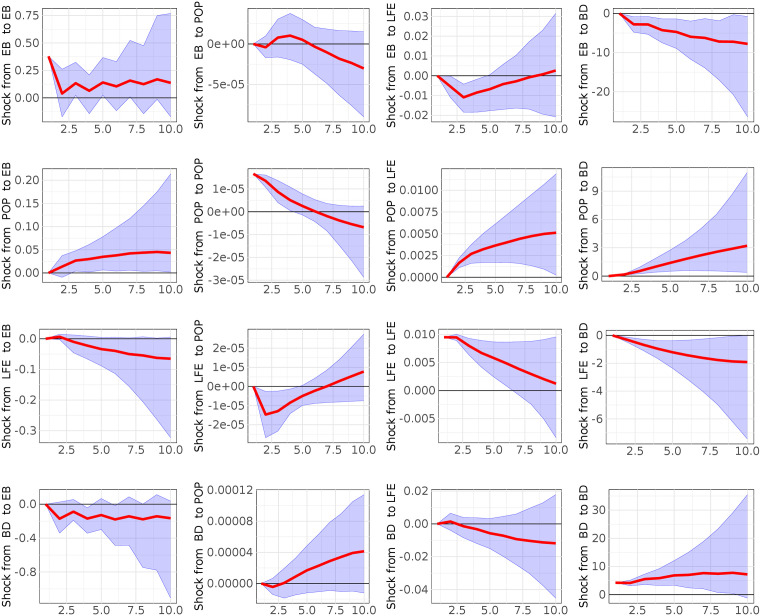
Computed Impulse Responses of population growth (POP), Ease of Doing Business (EB), Life expectancy at birth (LFE) and disease burden (BD) for a horizon of 10 years. Grey lines denote 5^th^ and 95^th^ credible sets (model 1).

**Fig 3:**
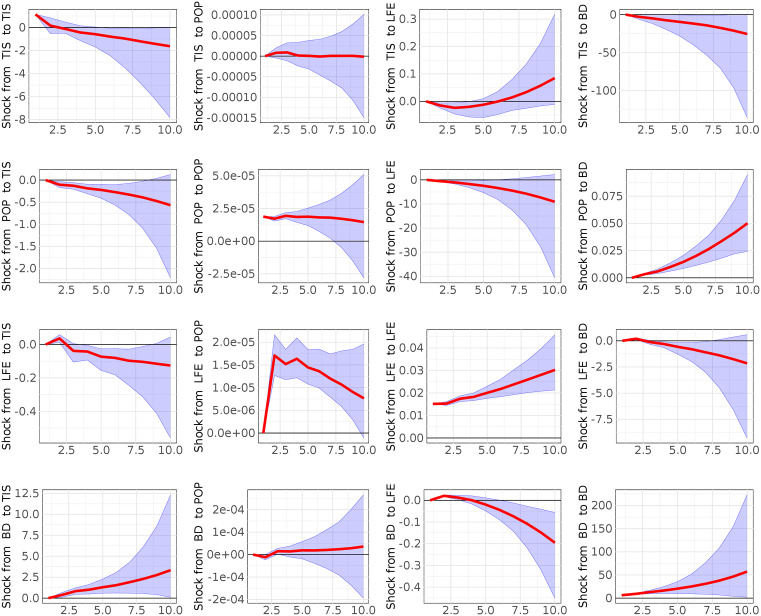
Computed Impulse Responses of population growth (POP), Trade in services (TIS), Life expectancy at birth (LFE) and disease burden (BD) for a horizon of 10 years. Grey lines denote 5^th^ and 95^th^ credible sets (model 2).

**Fig 4:**
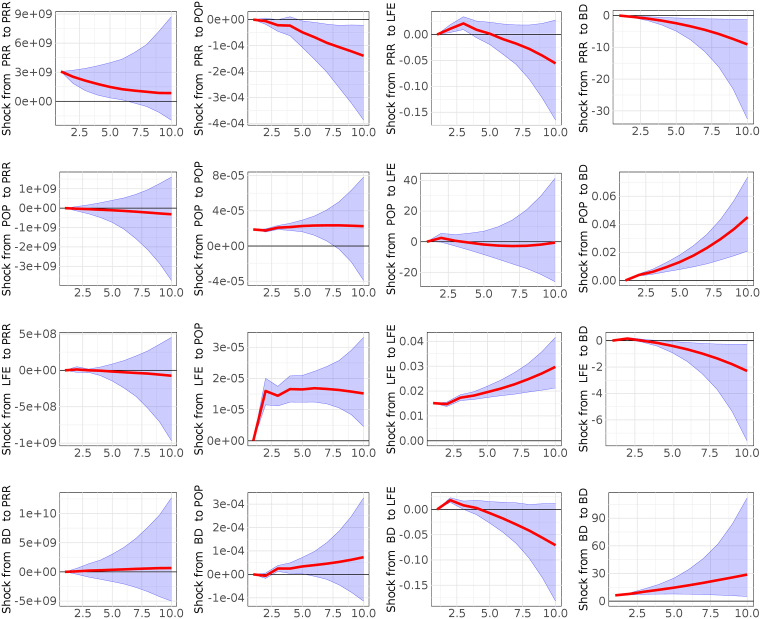
Computed Impulse Responses of population growth (POP), Personal Remittance Received (PRR), Life expectancy at birth (LFE) and disease burden (BD) for a horizon of 10 years. Grey lines denote 5^th^ and 95^th^ credible sets (model 3).

We interpret the results through the impulse response functions and the forecast variance decomposition functions based on the B-VAR framework to capture whether there exist significant relationships between our variables. The results of the forecast variance decomposition functions are shown in the supporting information (S1–S3 Figs). Figs 2–4 illustrate the impact of various shocks on the posterior density of the impulse response functions with central estimates shown as solid lines and credible bands visualized shaded areas that indicate the posterior coverage intervals corresponding to the 95% of the Bayesian credible sets obtained via 1000 Gibbs sampling iterations. The variables ordering (ORDER I) is as follows: a) EB, POP, LFE vs BD (model 1); b) TIS, POP, LFE vs BD (model 2); c) PRR, POP, LFE vs BD (model 3). We display the effect (one standard deviation) of a variable to the other variables for each B-VAR model until its effect becomes negligible (10 years) to capture both immediate and longer-term effects. If there is a reaction of one variable to an impulse in the other variable, then we can say that the latter is causal of the former. We perform the analysis for the period 2006–2018 due to data availability. The results from B-VAR model 1 shown in Fig 2 suggest that life expectancy at birth (LFE), population growth (POP) and wealth creation (Ease of doing Business-EB) are significant -drivers of the disease burden in Nigeria. As 10 percent shock to life expectancy at birth (LFE) leads to a reduction of disease burden of 40 percent after 7.5 years (BD) (third row, fourth column- LFE to BD). This finding implies that improvements in living conditions and healthcare access, reflected in higher life expectancy, are associated with reduced burden in health outcomes. A similar but more persistent effect is observed for wealth creation (EB) (first row, fourth column-EB to BD). A 1% percent increase in wealth creation (EB) is followed by a 10 percent decline in disease burden (BD). This effect remains significant for about 10 years. This result highlights the long-term influence of economic opportunity on public health, likely through increased access to healthcare and improved living standards. Finally, the response of disease burden (BD) to population growth (POP) is significant and positive (second row, fourth column-POP to BD). A 10 percent increase in population growth leads to a 30% percent rise in disease burden, suggesting that population pressures may contribute to the spread of illness by increasing contact rates and exposure.

Figs 3 and 4 report results from B-VAR models 2 and 3, which substitute the Ease of Doing Business (EB) variable with Trade in Services (TIS) and Personal Remittances Received (PRR), respectively. In both models, these alternative measures of wealth creation continue to show a statistically significant and negative relationship with disease burden. These findings confirm the central role of economic factors in shaping health outcomes in Nigeria, regardless of how wealth creation is operationalized. Moreover, to examine if the ordering of the variables changes the results of the impulse response functions, we computed the impulse response functions with the following variables ordering:

**ORDER II**: a) BD, POP, LFE vs EB (model 1); b) BD, POP, LFE vs TIS (model 2); c) BD, POP, LFE vs PRR (model 3)- **ORDER III**: a) EB, BD, LFE vs POP (model 1); b)TIS, BD, LFE vs POP (model 2); c)PRR, BD, LFE vs POP (model 3). **ORDER IV:** a) EB, POP, BD vs LFE (model 1); b) TIS, POP, BD vs LFE (model 2); c) PRR, POP, BD vs LFE (model 3)The results show that the ordering of the variables does not affect our analysis (S2 File Impulse response functions with the reverse ordering in the supporting information).

In addition, as robustness check we performed the sensitive analysis (shown in the supporting information in S1 File- Sensitivity analysis on Minnesota Prior Hyperparameters) changing the values of hyperparameters $(\lambda_{\text{1}}$, $\lambda_{\text{2}}$, $\lambda_{\text{3}},\lambda_{\text{4}}$) to analyze: a) whether the relationship between two variables, as indicated by the IRFs shocks, consistently changes or remains stable across the majority of the hyperparameter space explorations; b) which hyperparameter most significantly influences any observed sign changes and at which value these changes occur. Using a grid exploration we found that in the model 1 (EB case), model 2 (TIS case) and model 3 (PRR case) the impact respectively of EB on BD (99%), TIS on BD (100%), and PRR on BD (100%) is notably stronger than the reverse BD on EB (68%), BD on TIS (0%), BD on PRR (0). Moreover, we observe a significant impact of LFE on BD (93% model 1; 90% model 2–3) and POP on BD (100% model 1; 100% model 2–3) in all three models. As last step, our analysis confirmed that the hyperparameter $\lambda_{\text{1}}$, is demonstrably the most influence parameter. A key finding is that a smaller $\lambda_{\text{1}}$ value, as it is just set in our study, typically produces larger Impulse Response Function (IRF) values for the effects of Ease of Doing Business (EBD), Trade in Services (TIS), and Personal Remittances Received (PRR) on Disease Burden (BD). Furthermore, this smaller $\lambda_{\text{1}}$consistently leads to more pronounced IRF values for the impacts of Population Growth (POP) and Life Expectancy (LFE) on Disease Burden (BD), a pattern observed across all three distinct models (EBD, TIS, and PRR cases). Overall, the findings of sensitivity analysis confirm our manuscript’s results.

Finally, the forecast error variance decompositions, reported in the supporting information (S1–S3 Figs), reinforce the findings from the impulse response analysis by showing that a substantial proportion of the variance in future disease burden is explained by shocks to life expectancy at birth, population growth, and economic indicators. These results collectively support the conclusion that improvements in health and reductions in disease burden are closely tied to gains in population wellbeing and economic capacity.

## 4. Discussion

The findings of the study make a significant contribution to the literature focused on Nigeria by establishing and quantifying the dynamic relationships between key socioeconomic and health indicators with the burden disease. The results demonstrate that economic factors (EB, TIS, PRR) and public health indicators (LFE, POP) are not just correlated with disease burden but are significant drivers of it.

Specifically, we find a long-term influence of economic conditions (EB, TIS, PRR) and public health (LFE) on the well-being of the Nigerian population (BD). The negative relationship between wealth creation (EB, TIS, PRR) and disease burden (BD) suggests that economic empowerment and improved living standards are crucial for enhancing public health. Similarly, the finding that a rise in life expectancy (LFE) reduces disease burden (BD) underscores the social benefits of investing in healthcare, sanitation, and education. Conversely, the positive relationship between population growth (POP) and disease burden (BD) points out potential social challenges, such as the strain on healthcare infrastructure and increased risk of disease transmission in densely populated areas.

Overall, the findings provide critical evidence for policymakers in Nigeria. The clear link between wealth creation (EB, TIS, PRR) and reduced disease burden (BD) suggests that policies aimed at fostering economic growth and improving the business environment will have a direct, positive impact on public health. Policymakers should consider integrating health objectives into economic development strategies. Additionally, the results concerning life expectancy (LFE) and disease burden (BD) underscore the importance of continued investment in healthcare access, medical infrastructure, and public health programs. The negative impact of population growth on health outcomes suggests a need for strategic planning to manage population pressures, potentially through public health campaigns on family planning, and by ensuring that the growth of healthcare services follows population expansion.

Finally, the results offer a data-driven guide for resource allocation and program design. The persistent and significant effect of economic indicators (EB, TIS, PRR) on disease burden (BD) suggests that development organizations should not only focus on traditional health interventions but also on initiatives that promote economic opportunity and improve living standards. For example, programs that provide financial literacy, micro-loans, or business training could be combined with public health services. The results also provide useful insights into policies aimed at simplifying business processes (improving EB) or promoting trade and remittances (TIS, PRR) as these efforts can have a long-term impact on the health of the population.

## 5. Forecasting assessment

In this section, we assess the B-VAR model ability to fit the data using a Minnesota prior (our benchmark) by comparing it with an unrestricted VAR model and a B-VAR model based on Normal-Wishart prior. We also consider the B-VAR model specification on the Normal-Wishart prior because it yields to an analytical posterior without assuming the posterior independence between equations, and the variance-covariance matrix ∑ as known (with fixed and diagonal residuals).

To this end, since we start from a limited sample size, we drop the last two observations and using both the Root Mean Square Errors (RMSE) and the Mean Absolute Errors (MAE) criteria we ascertain which model shows the smallest forecast errors. We use the MAE and RMSE metrics together to diagnose the variation in the errors in the forecast for the following reasons:

- For both the MAE and RMSE the range goes from 0 to ∞ and a lower value of both criteria is better.
- The RMSE is a quadratic scoring square rule that gives relatively high weight to large errors.
- The MAE is a linear score that weighs the average equally with the individual differences. In this way it is possible to avoid the problem of negative and positive forecasts (absolute values) but sometimes it is difficult to separate a large error from a small one.

Thus, the RMSE may be larger or equal to the MAE: if the errors are of the same magnitude, we have RMSE equal to MAE; otherwise, if occur significant differences in the sample that means a greater “variance” in the individual errors in the sample we have RMSE larger than MAE.

In Tables 1–3 we report the RMSE, and MAE of each variable inserted in the models that we estimated to analyze the disease burden in Nigeria. Specifically in Table 1 we report the results inserted in the models Ease of Doing Business index (EB) while in Table 2 and 3 the results are shown for the models estimated with respectively Trade in Services (TS) and Personal Remittance Received (PRR).

**Table 1:** Forecasting VAR vs B-VAR model with Ease of Doing Business index.

	Variable	RMSE	MAE
Model			
Standard VAR	BD EB LFE POP	0.28 0.11 0.03 0.12	0.27 0.12 0.03 0.11
Minnesota prior	BD EB LFE POP	0.10 0.06 0.02 0.07	0.10 0.05 0.02 0.05
Normal-Wishart prior	BD EB LFE POP	0.25 0.07 0.03 0.08	0.24 0.06 0.04 0.10

**Table 2:** Forecasting VAR vs B-VAR model with Trade in Services (TIS).

	Variable	RMSE	MAE
Model			
Standard VAR	BD TIS LFE POP	0.26 0.10 0.05 0.10	0.25 0.09 0.03 0.11
Minnesota prior	BD TIS LFE POP	0.11 0.05 0.01 0.06	0.10 0.04 0.02 0.03
Normal-Wishart prior	BD TIS LFE POP	0.22 0.08 0.04 0.07	0.21 0.06 0.04 0.07

**Table 3:** Forecasting VAR vs B-VAR model with Personal Remittance Received (PRR).

	Variable	RMSE	MAE
Model			
Standard VAR	BD PRR LFE POP	0.25 0.11 0.02 0.08	0.23 0.12 0.04 0.09
Minnesota prior	BD PRR LFE POP	0.12 0.01 0.01 0.01	0.15 0.01 0.02 0.01
Normal-Wishart prior	BD PRR LFE POP	0.22 0.01 0.02 0.05	0.22 0.01 0.04 0.05

The results of the forecasting Nigerian disease burden of both RMSE and MAE are robust and similar underling that B-VAR with Minnesota prior, outperforms both unrestricted VAR model and the Normal Wishart BVAR model prior.

## 6. Conclusions and policy implications

This study investigates the leading causes of disease burden in Nigeria over the period 2006–2018. Since we analyze a sample limited in size due to data availability, we employ B-VAR models based on Minnesota prior that are theoretically grounded, easy to implement, and demonstrate a high accuracy both in fitting the data, and in estimating the impulse response functions and the forecast variance decomposition functions with the B-VAR models that are performing competitively compared to equivalent VAR models. Moreover, we estimate the impact of three different measures of wealth creation on disease burden in Nigeria to better understand the effects of wealth creation on that measure, with useful insights for policy recommendations.

The main result is that wealth creation expressed as “Ease of Doing Business”, “Trade in Services” and “Personal Remittance Received” has a great and significant impact on disease burden in Nigeria. Specifically, more wealth (a rise of “Ease of Doing Business”, “Trade in Services” and “Personal Remittance Received”) which increases people’s well-being can be translated into a lessened disease burden. At the same time, we find that life expectancy and population growth have a significant impact on disease burden. Our findings show that an increase of 10 percent of life expectancy at birth leads to a drop of 40 percent of disease burden. In this case a rise in life expectancy at birth is related to a better social welfare state that, among other things, can mitigate the effect of a pandemic.

Meanwhile, an increase of the population, ceteris paribus, has a positive effect on Nigeria disease burden but it also creates the conditions for a faster spread of infective diseases. This is not surprising: an increase of population leads to more social interaction between people and infectious diseases grow up. In summary, according to our empirical analysis we strongly recommended policy interventions and health strategies aimed to the promotion of higher income and hence more wealth creation in Nigeria.

Considering wealth benefits could result in favoring treatments for disease burden affecting individuals during their working life. The improved health status of the working individuals can also have positive effects on the economy of Nigeria by allowing the individuals to remain in work longer and maintain their labor supply.

In this context a larger population can be an advantage for Nigeria because it results in a “demographic dividend” that accelerates socio-economic growth resulting in a decline of both country’s mortality and fertility rate and then a change in the population age structure. From a national government’s perspective, funding interventions that increase wealth, income, and the patients’ ability to work set a virtuous cycle of “better health” which, in turn, could increase the total resources available and partly help dealing with the public deficit of Nigeria.

Moreover, government measures should prioritize investments in family planning-fostering a gender gender-equitable environment that empowers women to freely determine the number, timing, and spacing of their children. Additionally, efforts should focus on improving child health, and survival, alongside significant investment in education especially secondary education for girls [47,48]. In this way, ceteris paribus, it is possible to improve life expectancy at birth and reduce infant mortality. In a medium-term perspective, these plans have the potential advantage to equipping young women with effective skills for the labor force, thereby increasing their labor productivity and contributing to wealth creation. Thus, policy initiatives aimed at creating jobs, encouraging foreign investments, promoting exports of locally manufactured goods, and breaking the cycle of poverty, should be prioritized by the government and policy makers as a matter of urgency to achieve both economic growth and a demographic dividend by 2050. Effective resource management will contribute to improving patient welfare and may translate into a reduced disease burden, as well as cost savings in other sectors such as education and social care. We therefore conclude that the best health policies targeted at enhancing the wealth creation process will remain central to policy debate, making this work important from both economic and public policy perspectives. All in all, our findings, derived from an evidence-based approach, offer valuable insights for informing healthcare policy decisions. However, it’s crucial to acknowledge that these aggregated variables cannot be directly translated into specific policy instruments. Devising or evaluating a particular healthcare policy would require more detailed, micro-level investigations.

This study also has some limitations, primarily due to its scope: it covers a relatively short period (2006–2018) and includes only one country. To enhance the robustness of our results and generalize ther policy implications, future research should aim for larger datasets, incorporating longer time series or a greater number of countries. Finally, while B-VAR model is the best choice for this paper, alternatives or extensions for future research, even if less suitable for the current data limitations or research scope may be: Structural VAR (SVAR) Models: if future research allows for more data or if specific theoretical economic relationships become clearer in such a way to impose economic restrictions to identify structural shocks and their propagation mechanisms. While the current aim of our paper is to avoid a-priori restrictions, a future study might focus on specific policy interventions where identifying structural shocks is crucial. This would require careful justification and identification strategies. For example:

- Panel VAR Models: If we collect data for several Nigerian states or other similar developing countries. This would allow us to analyze common dynamic relationships across different states while accounting for heterogeneity.
- Time-Varying Parameter VAR (TVP-VAR) Models: For long-term analysis, economic and social relationships often there could be no static. TVP-VAR models allow the coefficients to evolve over time, capturing potential changes in the dynamic relationships between the variables due to structural breaks, policy shifts, or other evolving factors.
- Non-linear VAR Models: The relationships between disease burden, wealth, life expectancy, and population growth might not always be linear. Future research if we use longer time series could explore non-linear VAR extensions (e.g., Threshold VAR, Smooth Transition VAR) to capture potential thresholds or regime-switching behaviors in these dynamics.

These alternative approaches offer different strengths and complexities. Their suitability will depend on the specific research questions, data availability, and theoretical underpinnings of future studies. For the current study, however, B-VAR remains one of the most appropriate and robust choices given our aims and data constraints.

## Supporting information

S1 Fig FEVDs model 1 (EB model). (DOCX)

S2 Fig FEVDs model 2 (TIS model). (DOCX)

S3 Fig FEVDs model 3 (PRR model). (DOCX)

S1 File Sensitivity analysis on Minnesota Prior Hyperparameters. (DOCX)

S2 File Impulse response functions with the reverse ordering. (DOCX)

## Abbreviations

B-VAR model: Bayesian Vector Autoregressive Model
BD: Disease Burden
DALYs: Disability Adjusted Life Years
EB: Ease of doing business
FDI: Foreign Direct Investments
FEVDs: Forecast error decomposition functions
IRFs: Impulse Response Functions
GBD: Global Burden of Disease
LFE: Life expectancy at birth
MAE: Mean absolute errors
POP: Population growth
PRR: Personal remittance received
QAYLs: Quality adjusted life years
RMSE: Root mean square errors
SVAR: Structural VAR
TIS: Trade in services
TVP-VAR: Time-Varying Parameter VAR
VAR model: Vector autoregressive model
YLLs: Number of years of life lost
YLDs: Loss of Health.
