# Supplementary material for: Wealth creation and disease burden: Evidence from Nigeria based on a Bayesian-VAR approach
Source: PLoS One. 2025 Nov 10;20(11):e0334709. doi: 10.1371/journal.pone.0334709 (PMC12599923; doi:10.1371/journal.pone.0334709)
Supplement: S1 Fig — (DOCX) [file pone.0334709.s001.docx]

**Supplementary materials**

Below the Graphs of forecast variance decomposition functions (FEVDs) relative to the B-VAR model (EB case) just discussed in the manuscript.

**S1 Figure- FEVDs model 1 (EB model)**

**
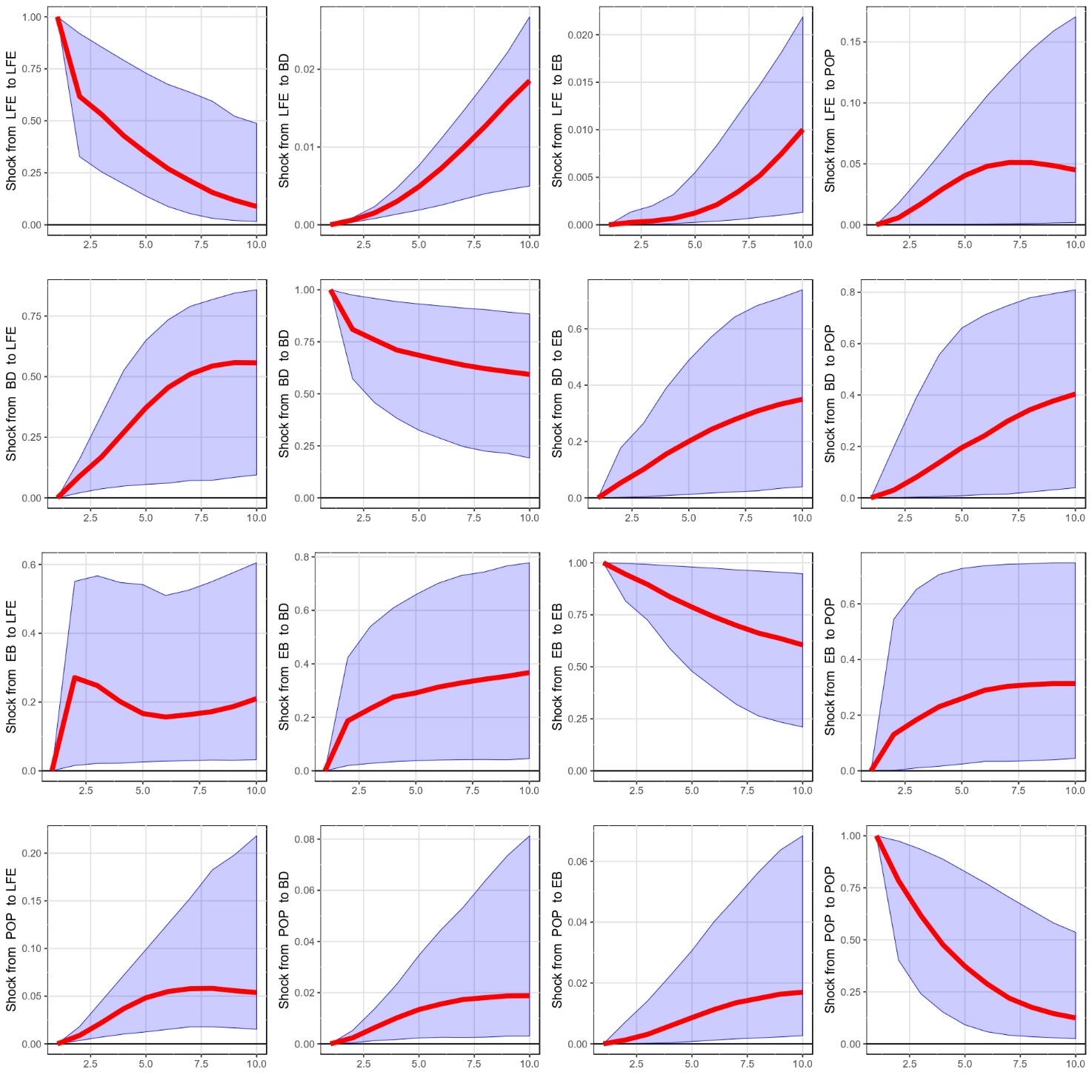
**
