# Supplementary material for: Wealth creation and disease burden: Evidence from Nigeria based on a Bayesian-VAR approach
Source: PLoS One. 2025 Nov 10;20(11):e0334709. doi: 10.1371/journal.pone.0334709 (PMC12599923; doi:10.1371/journal.pone.0334709)
Supplement: S1 File — (DOCX) [file pone.0334709.s004.docx]

**Supplementary materials**

**S1 file-Sensitivity analysis on Minnesota Prior Hyperparameters**

To perform the sensitivity analysis on Minnesota prior hyperparameters we use a grid exploration of the four hyperparameters $\lambda_{1}$, $\lambda_{2}$, $\lambda_{3}, \lambda_{4}$ (following the original R package BMR by Keith O'Hara that is based on the work of Canova 2007, Koop and Korobilis 2010). The B-VAR estimation with the Minnesota prior requires a total of 4 hyperparameters, with $\lambda_{4}$ (the lag decay) usually fixed, that influence how the model fits the original data.

The hyperparameters have the following meaning (Koop and Korobilis 2010):

- $\lambda_{1}$(Tightness of the AR coefficients):
  This determines how strongly we believe that most of the coefficients are close to zero - meaning that, by default, the model expects each variable will be mostly influenced by its own past, not by other variables of the model. Smaller   $\lambda_{1}$ means more belief that coefficients are near zero (more “shrinkage” i.e.  less influence from the past values of given variables).

  $\lambda_{2}$(Cross-variable Prior Tightness):
  This controls how much we believe the coefficients linking different variables with cross-correlation are close to zero. Smaller $\lambda_{2}$ indicates a stronger prior belief that variables do not influence each other much.
- $\lambda_{3}$(Observation Variance/Individual Variance Scaling):
  This controls how much we think the variability (uncertainty) of different variables varies. It affects how the prior re-scales the variances of the coefficients for different variables also exogenous.
- $\lambda_{4}$(Linear Decay or Harmonic Decay):
  This governs how quickly we expect the influence of past observations to decrease as they get older. It often follows a harmonic decay pattern, meaning that coefficients for more distant lags are shrunk more strongly. Smaller $\lambda_{4}$ implies a faster decay — the model expects recent past values to be much more important than older ones.

We expect a different response of the fitted B-VAR model (**model 1**: BD vs EB, POP, LFE; **model 2**: BD vs TIS, POP, LFE; **model 3**: BD vs PRR, POP, LFE) to each of the hyperparameters, and in particular to the $\lambda_{1}$ as smaller values imply more importance to the selected prior that outweighs any information in the data. $\lambda_{2}$ controls the cross-variable prior tightness a value that allows to weight the influence of the cross-variables in the prior model. $\lambda_{3}$ controls the influence of exogenous shocks while $\lambda_{4}$the decay of the lags, often fixed during simulation: reducing de facto the number of effective hyperparameters to 3. We investigated the response of the three B-VAR models (**model 1**: EB case; **model 2**: TIS case; **model 3**: PRR case) in the hyperparameters space (Koop and Korobilis 2010):

$\lambda_{1}$ in (0.001 , .., 1), with 10 equal steps

$\lambda_{2}$ in (0.1 ,..,  4),  with 5 equal steps

$\lambda_{3}$ in (0.1,..,  4), with  5 equal steps

$\lambda_{4}$ in (0.1,..,  4), with 5 equal steps

We ran a total of 10*5*5*5 = 1250 steps, allocating more steps to $\lambda_{1}$ due to its anticipated greater influence in the prior Minnesota (we assume that the prior beliefs are informative). Each step has 1000 random realization of the priors (Gibbs sampling).

We chose the intervals with enough room to investigate the space of hyperparameters, with in particular $\lambda_{1}$ that going from the smallest value 0.001 to the largest value 1 has a ratio of 1 / 0.001 = 1000, a difference on the shrinkage that is enough to check the sensitivity of the model to the hyperparameter and to confirm the findings. We apply the same logic but with less steps to the other hyperparameters balancing computing time with accuracy in the exploration of the solution stability.

The sensitivity tests were conducted as follows:

To evaluate how the variation of hyperparameters affect the dynamics of the Impulse Response Functions (IRFs), we estimated the three B-VAR models just discussed above for each hyperparameter set across all possible variable combinations (e.g., the impact of “POP” on “BD”, “EB” on “BD” and so on). For every shock of IRFs, we then assessed the statistical significance of the resulting relationship as follows (Figure 1):

A) Significant: both confidence bands lie in the same quadrant, or they change direction after lag > 5 (at least 5 years of both positive/negative shock (+,+; - -) over a period of 10 years)).

B)  Non significant: confidence bands lie in different quadrants (e.g +, -; -, +)


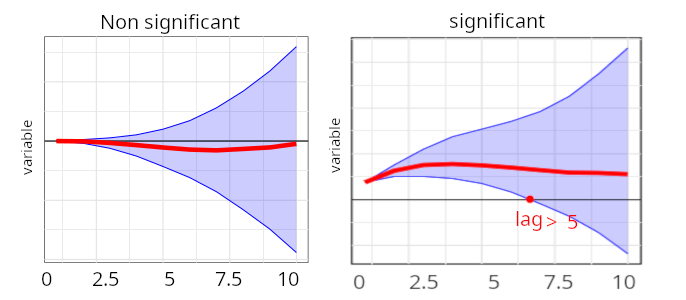


**Figure 1**. IRFs test schema: on the left the confidence bands are sitting in different quadrants, on the right there are many lags (> 5) that are in the same quadrant

Next, we conducted a test to determine the significance of the IRFs relationships across the entire hyperparameter space exploration. Specifically, our aim was to assess:

1. Whether the relationship between two variables, as indicated by the IRFs shocks, consistently changes or remains stable across the majority of the 1250 steps in the hyperparameter space explorations (*test 1*)- **Figure 2**.
2. Identifying which hyperparameter most significantly influences any observed sign changes and at which value these changes occur (*test 2*)- **Table 1**.

As the exploration is large, many analyses are possible. We summarized the findings of the *test 1* for the three B-VAR models (**model 1**: EB case; **model 2**: TIS case; **model 3**: PRR case) with the following heatmap (**Figure 2**).


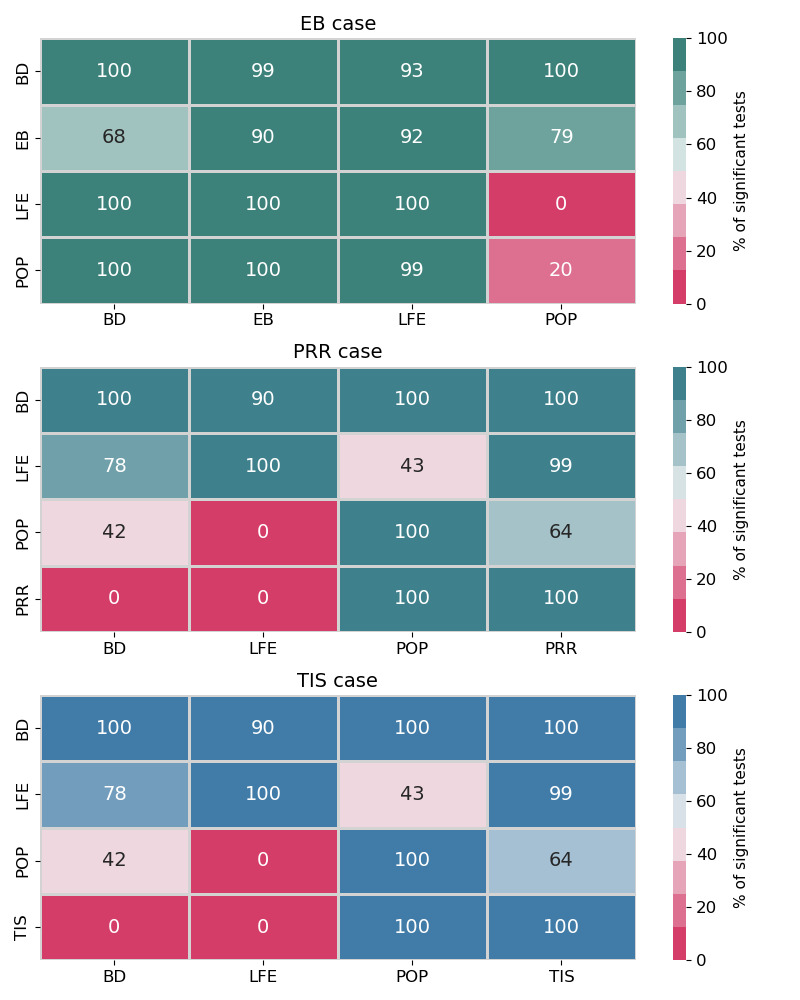


**Figure 2**.  Summary of sensitivity tests with number of significant tests in percentage. Below the threshold of 50 % the relationship is non-significant.

In Figure 2 we summarized the significance of the IRFs tests, assessing how many tests pass (in percentage) for a specific shock. We see that only a fraction of the relationships is always passing the test (value 100%) or never passing the test (value 0%) and often these relationships are non-symmetric.

For instance, in the **model 1** (EB case) the impact of EB on BD (99%) is notably stronger than the reverse BD on EB (68%). Moreover, we observe a significant impact of LFE on BD (93%) and POP on BD (100%).

Similarly, in the **model 2** (TIS case) the respective impacts TIS on BD (100%), LFE on BD (90%) and POP on BD (100%) are all significant.

Finally, in the **model 3** (PRR case) we find a significant impact of PRR on BD (100%), LFE on BD (90%) and POP on BD (100%)

Collectively, these findings reinforce the main conclusions of the manuscript.

To investigate which hyperparameter is more influential (*test 2*), we show the results of the IRFs relative to the three B-VAR models (**model 1**-Table 1a, **model 2**-Table 1b, **model 3**-Table 1c) in **Table 1** (a,b,c). This table details the number of significant Impulse Response Function (IRFs) tests for each hyperparameter value across all possible combinations. We performed a total of 1250 hyperparameter explorations. Since $\lambda_{1}$ had 10 individual steps, each of its values could appear a maximum of 125 times (10*125=1250). For $\lambda_{2}$, $\lambda_{3}$, and $\lambda_{4}$, which each had 5 steps, every value could appear up to 250 times (5*250=1250).

We can summarize **Table 1** as follows:

The hyperparameter $\lambda_{1}$, which governs the tightness of the AR coefficients in the prior, is demonstrably the most relevant parameter, consistent with our expectations.

Usually, higher Impulse Response Function (IRFs) values (highlighted in yellow in the Table 1) for the impacts of Ease of Doing Business (EB: **var_0**), Trade in Services (TIS: **var_0**), and Personal Remittances Received (PRR: **var_0**) on Disease Burden (BD: **var_1**) are obtained when $\lambda_{1}$ is set small as in our study. Furthermore, setting small the value of $\lambda_{1}$ leads to higher IRF values (highlighted in yellow in the Table 1, , we select only the first two columns because in our B-VAR model we set $\lambda_{1}$ small) for the impacts of Population Growth (POP: **var_0**) and Life Expectancy (LFE: **var_0**) on Burden Disease (BD: **var_1**) across all three models (EB, TIS, and PRR cases).

A sensitivity dependency is also observed for the hyperparameters $\lambda_{2}$ and $\lambda_{3}$(the highest values are highlighted in yellow in the Table 1, we select only the first two columns because in our B-VAR model we set $\lambda_{2}$ greater than zero and λ3 is set close to zero). Their influence is relevant in the relationships of EB (**var_0**), TIS (**var_0**), PRR (**var_0**) versus BD (**var_1**), as well as POP (**var_0**) and LFE (**var_0**) on BD (**var_1**), across the three models 1, 2, and 3 (**Table 1a**, **1b**, and **1c**).

 In summary, the $\lambda_{1}$ showing the highest values of the IRFs relationships just discussed above is confirmed to be the most important hyperparameter for the dataset as it can influence the significance of several cases: EB, TIS, PRR vs BD, and, POP, LFE vs BD. In all other cases the changes of number of passed tests in function of the hyperparameters are nonsignificant or very small and can be seen as statistical fluctuations.

| **var_0**  **X** | **var_1**  **Y** | $\boldsymbol{\lambda}_{\boldsymbol{1}}$ **= 0.001** | $\boldsymbol{\lambda}_{\boldsymbol{1}}$ **= 0.112** | $\boldsymbol{\lambda}_{\boldsymbol{1}}$ **= 0.223** | $\boldsymbol{\lambda}_{\boldsymbol{1}}$ **= 0.334** | $\boldsymbol{\lambda}_{\boldsymbol{1}}$ **= 0.445** | $\boldsymbol{\lambda}_{\boldsymbol{1}}$ **= 0.556** | $\boldsymbol{\lambda}_{\boldsymbol{1}}$ **= 0.667** | $\boldsymbol{\lambda}_{\boldsymbol{1}}$**= 0.778** | $\boldsymbol{\lambda}_{\boldsymbol{1}}$ **= 0.889** | $\boldsymbol{\lambda}_{\boldsymbol{1}}$ **= 1.000** |
| --- | --- | --- | --- | --- | --- | --- | --- | --- | --- | --- | --- |
| **BD** | **EB** | 10 | 2 | 0 | 0 | 0 | 0 | 0 | 0 | 0 | 0 |
|  | **LFE** | 10 | 0 | 0 | 0 | 0 | 0 | 0 | 0 | 0 | 0 |
| **BD** | **POP** | 2 | 0 | 0 | 0 | 0 | 0 | 0 | 0 | 0 | 0 |
| **BD** | **BD** | 125 | 125 | 125 | 125 | 125 | 125 | 125 | 125 | 99 | 99 |
| **EB** | **BD** | 137 | 115 | 118 | 116 | 110 | 27 | 21 | 25 | 21 | 21 |
|  | **EB** | 125 | 0 | 0 | 0 | 0 | 0 | 0 | 0 | 0 | 0 |
|  | **LFE** | 19 | 22 | 11 | 3 | 4 | 2 | 2 | 1 | 1 | 0 |
|  | **POP** | 12 | 12 | 2 | 1 | 1 | 0 | 0 | 0 | 0 | 0 |
| **LFE** | **POP** | 25 | 25 | 25 | 25 | 25 | 25 | 25 | 25 | 25 | 25 |
| **LFE** | **LFE** | 30 | 30 | 60 | 60 | 50 | 50 | 50 | 50 | 50 | 50 |
|  | **EB** | 0 | 0 | 19 | 25 | 25 | 25 | 25 | 25 | 25 | 25 |
| **LFE** | **BD** | 100 | 100 | 80 | 80 | 70 | 70 | 60 | 55 | 55 | 55 |
| **POP** | **LFE** | 100 | 70 | 70 | 70 | 40 | 35 | 12 | 12 | 10 | 7 |
| **POP** | **BD** | 25 | 25 | 25 | 10 | 10 | 10 | 10 | 10 | 10 | 10 |
| **POP** | **EB** | 20 | 10 | 0 | 0 | 0 | 0 | 0 | 0 | 0 | 0 |
| **var_0** | **var_1** | $\boldsymbol{\lambda}_{\boldsymbol{2}}$ **= 0.100** | $\boldsymbol{\lambda}_{\boldsymbol{2}}$ **= 1.075** | $\boldsymbol{\lambda}_{\boldsymbol{2}}$ **= 2.050** | $\boldsymbol{\lambda}_{\boldsymbol{2}}$ **= 3.025** | $\boldsymbol{\lambda}_{\boldsymbol{2}}$ **= 4.000** |  |  |  |  |  |
| **BD** | **EB** | 0 | 0 | 1 | 1 | 0 |  |  |  |  |  |
|  | **LFE** | 240 | 234 | 135 | 138 | 130 |  |  |  |  |  |
| **BD** | **POP** | 100 | 100 | 10 | 10 | 0 |  |  |  |  |  |
| **BD** | **BD** | 240 | 240 | 105 | 105 | 0 |  |  |  |  |  |
| **EB** | **BD** | 81 | 75 | 85 | 80 | 77 |  |  |  |  |  |
|  | **EB** | 25 | 25 | 25 | 25 | 25 |  |  |  |  |  |
|  | **LFE** | 25 | 17 | 18 | 20 | 15 |  |  |  |  |  |
|  | **POP** | 49 | 49 | 45 | 42 | 46 |  |  |  |  |  |
| **LFE** | **POP** | 250 | 250 | 250 | 250 | 250 |  |  |  |  |  |
| **LFE** | **LFE** | 1 | 0 | 0 | 0 | 2 |  |  |  |  |  |
|  | **EB** | 20 | 20 | 19 | 19 | 19 |  |  |  |  |  |
| **LFE** | **BD** | 90 | 90 | 78 | 78 | 79 |  |  |  |  |  |
| **POP** | **LFE** | 1 | 0 | 0 | 0 | 2 |  |  |  |  |  |
|  | **POP** | 200 | 200 | 199 | 198 | 197 |  |  |  |  |  |
| **POP** | **EB** | 2 | 2 | 19 | 19 | 17 |  |  |  |  |  |
| **POP** | **DB** | 90 | 50 | 69 | 18 | 17 |  |  |  |  |  |
| **var_0** | **var_1** | $\boldsymbol{\lambda}_{\boldsymbol{3}}$ **= 0.100** | $\boldsymbol{\lambda}_{\boldsymbol{3}}$ **= 1.075** | $\boldsymbol{\lambda}_{\boldsymbol{3}}$ **= 2.050** | $\boldsymbol{\lambda}_{\boldsymbol{3}}$ **= 3.025** | $\boldsymbol{\lambda}_{\boldsymbol{3}}$ **= 4.000** |  |  |  |  |  |
| **BD** | **EB** | 1 | 0 | 0 | 1 | 0 |  |  |  |  |  |
|  | **LFE** | 25 | 23 | 26 | 25 | 28 |  |  |  |  |  |
| **BD** | **POP** | 5 | 3 | 6 | 5 | 18 |  |  |  |  |  |
| **BD** | **LFE** | 5 | 8 | 6 | 9 | 11 |  |  |  |  |  |
| **EB** | **BD** | 84 | 64 | 88 | 85 | 77 |  |  |  |  |  |
|  | **EB** | 25 | 25 | 25 | 25 | 25 |  |  |  |  |  |
|  | **LFE** | 17 | 17 | 21 | 18 | 22 |  |  |  |  |  |
|  | **POP** | 53 | 53 | 49 | 52 | 54 |  |  |  |  |  |
| **LFE** | **POP** | 50 | 50 | 50 | 250 | 250 |  |  |  |  |  |
| **LFE** | **EB** | 20 | 10 | 10 | 5 | 6 |  |  |  |  |  |
| **LFE** | **DB** | 85 | 80 | 100 | 95 | 90 |  |  |  |  |  |
| **LFE** | **LFE** | 200 | 200 | 150 | 100 | 100 |  |  |  |  |  |
| **POP** | **LFE** | 1 | 0 | 1 | 1 | 0 |  |  |  |  |  |
|  | **POP** | 199 | 199 | 197 | 200 | 199 |  |  |  |  |  |
| **POP** | **DB** | 50 | 40 | 30 | 30 | 40 |  |  |  |  |  |
| **POP** | **EB** | 20 | 10 | 2 | 2 | 1 |  |  |  |  |  |
| **var_0** | **var_1** | $\boldsymbol{\lambda}_{\boldsymbol{4}}$ **= 0.100** | $\boldsymbol{\lambda}_{\boldsymbol{4}}$**= 1.075** | $\boldsymbol{\lambda}_{\boldsymbol{4}}$ **= 2.050** | $\boldsymbol{\lambda}_{\boldsymbol{4}}$ **= 3.025** | $\boldsymbol{\lambda}_{\boldsymbol{4}}$ **= 4.000** |  |  |  |  |  |
| **BD** | **EB** | 0 | 1 | 0 | 1 | 0 |  |  |  |  |  |
|  | **LFE** | 34 | 33 | 35 | 36 | 39 |  |  |  |  |  |
| **BD** | **BD** | 5 | 13 | 6 | 15 | 18 |  |  |  |  |  |
| **BD** | **POP** | 15 | 18 | 6 | 19 | 21 |  |  |  |  |  |
| **EB** | **BD** | 32 | 30 | 32 | 36 | 38 |  |  |  |  |  |
|  | **EB** | 25 | 25 | 25 | 25 | 25 |  |  |  |  |  |
|  | **LFE** | 16 | 14 | 17 | 19 | 29 |  |  |  |  |  |
|  | **POP** | 54 | 52 | 51 | 53 | 51 |  |  |  |  |  |
| **LFE** | **POP** | 50 | 50 | 50 | 50 | 50 |  |  |  |  |  |
| **LFE** | **DB** | 25 | 14 | 12 | 10 | 6 |  |  |  |  |  |
| **LFE** | **EB** | 5 | 4 | 0 | 0 | 0 |  |  |  |  |  |
| **LFE** | **LFE** | 54 | 43 | 3 | 0 | 0 |  |  |  |  |  |
| **POP** | **LFE** | 0 | 1 | 0 | 1 | 1 |  |  |  |  |  |
|  | **POP** | 200 | 199 | 198 | 200 | 197 |  |  |  |  |  |
| **POP** | **EB** | 0 | 2 | 5 | 1 | 1 |  |  |  |  |  |
| **POP** | **BD** | 45 | 54 | 33 | 12 | 1 |  |  |  |  |  |

**Table 1a**:  It shows the results of the model 1 (EB case), identifying which hyperparameters are most influential in causing sign changes in the Impulse Response Functions (IRFs) and at which specific values these directional shifts are observed.

| **var_0** | **var_1** | $\boldsymbol{\lambda}_{\boldsymbol{1}}$**= 0.001** | $\boldsymbol{\lambda}_{\boldsymbol{1}}$**= 0.112** | $\boldsymbol{\lambda}_{\boldsymbol{1}}$ **= 0.223** | $\boldsymbol{\lambda}_{\boldsymbol{1}}$**= 0.334** | $\boldsymbol{\lambda}_{\boldsymbol{1}}$**= 0.445** | $\boldsymbol{\lambda}_{\boldsymbol{1}}$**= 0.556** | $\boldsymbol{\lambda}_{\boldsymbol{1}}$**= 0.667** | $\boldsymbol{\lambda}_{\boldsymbol{1}}$**= 0.778** | $\boldsymbol{\lambda}_{\boldsymbol{1}}$**= 0.889** | $\boldsymbol{\lambda}_{\boldsymbol{1}}$**= 1.000** |
| --- | --- | --- | --- | --- | --- | --- | --- | --- | --- | --- | --- |
| **BD** | **TIS** | 0 | 3 | 0 | 0 | 0 | 0 | 0 | 0 | 0 | 0 |
|  | **LFE** | 25 | 25 | 25 | 25 | 24 | 21 | 17 | 12 | 7 | 7 |
| **BD** | **POP** | 2 | 0 | 0 | 0 | 0 | 0 | 0 | 0 | 0 | 0 |
| **BD** | **BD** | 125 | 125 | 125 | 125 | 125 | 125 | 125 | 125 | 99 | 99 |
| **TIS** | **BD** | 124 | 118 | 121 | 45 | 47 | 41 | 38 | 25 | 18 | 11 |
|  | **TIS** | 124 | 0 | 0 | 0 | 0 | 0 | 0 | 0 | 0 | 0 |
|  | **LFE** | 0 | 0 | 7 | 14 | 18 | 11 | 19 | 11 | 17 | 24 |
|  | **POP** | 5 | 4 | 8 | 2 | 1 | 1 | 0 | 0 | 1 | 0 |
| **LFE** | **POP** | 125 | 115 | 105 | 85 | 75 | 45 | 5 | 5 | 5 | 5 |
| **LFE** | **LFE** | 120 | 50 | 50 | 4 | 0 | 0 | 0 | 0 | 0 | 0 |
| **LFE** | **BD** | 97 | 84 | 52 | 32 | 0 | 0 | 0 | 0 | 0 | 0 |
| **LFE** | **TIS** | 40 | 34 | 0 | 0 | 0 | 0 | 0 | 0 | 0 | 0 |
| **POP** | **LFE** | 0 | 6 | 1 | 0 | 0 | 0 | 0 | 0 | 0 | 0 |
|  | **POP** | 0 | 0 | 109 | 125 | 125 | 125 | 125 | 125 | 125 | 125 |
| **POP** | **BD** | 70 | 70 | 15 | 15 | 0 | 0 | 0 | 0 | 0 | 0 |
| **POP** | **TIS** | 15 | 15 | 10 | 0 | 0 | 0 | 0 | 0 | 0 | 0 |
| **var_0** | **var_1** | $\boldsymbol{\lambda}_{\boldsymbol{2}}$ **= 0.100** | $\boldsymbol{\lambda}_{\boldsymbol{2}}$ **= 1.075** | $\boldsymbol{\lambda}_{\boldsymbol{2}}$ **= 2.050** | $\boldsymbol{\lambda}_{\boldsymbol{2}}$**= 3.025** | $\boldsymbol{\lambda}_{\boldsymbol{2}}$**= 4.000** |  |  |  |  |  |
| **BD** | **TIS** | 10 | 10 | 1 | 1 | 0 |  |  |  |  |  |
|  | **LFE** | 46 | 37 | 35 | 35 | 33 |  |  |  |  |  |
| **BD** | **POP** | 1 | 1 | 0 | 0 | 0 |  |  |  |  |  |
| **BD** | **BD** | 10 | 11 | 14 | 32 | 4 |  |  |  |  |  |
| **TIS** | **BD** | 101 | 100 | 102 | 88 | 76 |  |  |  |  |  |
|  | **TIS** | 25 | 25 | 25 | 25 | 25 |  |  |  |  |  |
|  | **LFE** | 25 | 19 | 14 | 22 | 25 |  |  |  |  |  |
|  | **POP** | 53 | 54 | 51 | 49 | 51 |  |  |  |  |  |
| **LFE** | **POP** | 71 | 74 | 82 | 76 | 79 |  |  |  |  |  |
| **LFE** | **TIS** | 27 | 34 | 23 | 11 | 11 |  |  |  |  |  |
| **LFE** | **DB** | 120 | 100 | 150 | 68 | 76 |  |  |  |  |  |
| **POP** | **LFE** | 1 | 0 | 0 | 0 | 3 |  |  |  |  |  |
|  | **POP** | 51 | 34 | 22 | 16 | 9 |  |  |  |  |  |
| **POP** | **DB** | 71 | 74 | 82 | 76 | 79 |  |  |  |  |  |
| **POP** | **TIS** | 25 | 12 | 0 | 0 | 0 |  |  |  |  |  |
| **var0** | **var1** | $\boldsymbol{\lambda}_{\boldsymbol{3}}$ **= 0.100** | $\boldsymbol{\lambda}_{\boldsymbol{3}}$ **= 1.075** | $\boldsymbol{\lambda}_{\boldsymbol{3}}$ **= 2.050** | $\boldsymbol{\lambda}_{\boldsymbol{3}}$ **= 3.025** | $\boldsymbol{\lambda}_{\boldsymbol{3}}$ **= 4.000** |  |  |  |  |  |
| **BD** | **TIS** | 1 | 0 | 0 | 1 | 0 |  |  |  |  |  |
|  | **LFE** | 45 | 41 | 46 | 41 | 47 |  |  |  |  |  |
| **BD** | **BD** | 13 | 11 | 0 | 0 | 0 |  |  |  |  |  |
| **BD** | **POP** | 11 | 2 | 3 | 1 | 2 |  |  |  |  |  |
| **TIS** | **BD** | 72 | 66 | 172 | 80 | 78 |  |  |  |  |  |
|  | **TIS** | 25 | 25 | 25 | 25 | 25 |  |  |  |  |  |
|  | **LFE** | 19 | 16 | 20 | 17 | 18 |  |  |  |  |  |
|  | **POP** | 18 | 18 | 17 | 14 | 12 |  |  |  |  |  |
| **LFE** | **POP** | 50 | 50 | 50 | 50 | 50 |  |  |  |  |  |
| **LFE** | **TIS** | 67 | 45 | 40 | 21 | 21 |  |  |  |  |  |
| **LFE** | **BD** | 124 | 124 | 122 | 110 | 67 |  |  |  |  |  |
| **LFE** | **LFE** | 120 | 120 | 120 | 120 | 120 |  |  |  |  |  |
| **POP** | **LFE** | 3 | 0 | 1 | 1 | 0 |  |  |  |  |  |
|  | **POP** | 201 | 191 | 199 | 203 | 202 |  |  |  |  |  |
| **POP** | **DB** | 91 | 81 | 79 | 63 | 42 |  |  |  |  |  |
| **POP** | **TIS** | 56 | 23 | 11 | 0 | 0 |  |  |  |  |  |
| **var_0** | **var_1** | $\boldsymbol{\lambda}_{\boldsymbol{4}}$ **= 0.100** | $\boldsymbol{\lambda}_{\boldsymbol{4}}$**= 1.075** | $\boldsymbol{\lambda}_{\boldsymbol{4}}$ **= 2.050** | $\boldsymbol{\lambda}_{\boldsymbol{4}}$ **= 3.025** | $\boldsymbol{\lambda}_{\boldsymbol{4}}$ **= 4.000** |  |  |  |  |  |
| **BD** | **TIS** | 0 | 1 | 0 | 1 | 0 |  |  |  |  |  |
|  | **LFE** | 30 | 30 | 24 | 24 | 24 |  |  |  |  |  |
| **BD** | **POP** | 20 | 11 | 23 | 0 | 0 |  |  |  |  |  |
| **BD** | **LFE** | 33 | 22 | 21 | 13 | 11 |  |  |  |  |  |
| **TIS** | **BD** | 14 | 13 | 14 | 12 | 14 |  |  |  |  |  |
|  | **TIS** | 25 | 25 | 25 | 25 | 25 |  |  |  |  |  |
|  | **LFE** | 13 | 11 | 19 | 21 | 31 |  |  |  |  |  |
|  | **POP** | 51 | 55 | 50 | 53 | 50 |  |  |  |  |  |
| **LFE** | **POP** | 50 | 50 | 50 | 50 | 50 |  |  |  |  |  |
| **LFE** | **BD** | 55 | 23 | 11 | 7 | 4 |  |  |  |  |  |
| **LFE** | **TIS** | 1 | 1 | 0 | 0 | 0 |  |  |  |  |  |
| **LFE** | **LFE** | 112 | 111 | 110 | 110 | 110 |  |  |  |  |  |
| **POP** | **LFE** | 0 | 1 | 0 | 2 | 1 |  |  |  |  |  |
|  | **POP** | 204 | 202 | 196 | 200 | 199 |  |  |  |  |  |
| **POP** | **DB** | 24 | 22 | 16 | 10 | 9 |  |  |  |  |  |
| **POP** | **TIS** | 1 | 0 | 1 | 1 | 0 |  |  |  |  |  |

**Table 1b**:  It shows the results of the model 2 (TIS case), identifying which hyperparameters are most influential in causing sign changes in the Impulse Response Functions (IRFs) and at which specific values these directional shifts are observed.

| **var_0** | **var_1** | $\boldsymbol{\lambda}_{\boldsymbol{1}}$**= 0.001** | $\boldsymbol{\lambda}_{\boldsymbol{1}}$**= 0.112** | $\boldsymbol{\lambda}_{\boldsymbol{1}}$ **= 0.223** | $\boldsymbol{\lambda}_{\boldsymbol{1}}$**= 0.334** | $\boldsymbol{\lambda}_{\boldsymbol{1}}$**= 0.445** | $\boldsymbol{\lambda}_{\boldsymbol{1}}$**= 0.556** | $\boldsymbol{\lambda}_{\boldsymbol{1}}$**= 0.667** | $\boldsymbol{\lambda}_{\boldsymbol{1}}$**= 0.778** | $\boldsymbol{\lambda}_{\boldsymbol{1}}$**= 0.889** | $\boldsymbol{\lambda}_{\boldsymbol{1}}$**= 1.000** |
| --- | --- | --- | --- | --- | --- | --- | --- | --- | --- | --- | --- |
| **BD** | **PRR** | 0 | 2 | 0 | 0 | 0 | 0 | 0 | 0 | 0 | 0 |
|  | **LFE** | 25 | 25 | 25 | 25 | 24 | 25 | 17 | 12 | 1 | 7 |
| **BD** | **BD** | 44 | 44 | 12 | 1 | 1 | 1 | 0 | 2 | 0 | 1 |
| **BD** | **POP** | 4 | 3 | 1 | 0 | 1 | 1 | 0 | 0 | 0 | 1 |
| **PRR** | **BD** | 116 | 113 | 80 | 61 | 38 | 30 | 24 | 27 | 22 | 21 |
|  | **PRR** | 125 | 0 | 0 | 0 | 0 | 0 | 0 | 0 | 0 | 0 |
|  | **LFE** | 0 | 0 | 3 | 7 | 8 | 11 | 10 | 16 | 17 | 22 |
|  | **POP** | 25 | 12 | 22 | 1 | 1 | 0 | 0 | 0 | 0 | 0 |
| **LFE** | **POP** | 12 | 12 | 15 | 15 | 0 | 0 | 0 | 0 | 0 | 0 |
| **LFE** | **PRR** | 1 | 0 | 1 | 1 | 2 | 7 | 1 | 0 | 1 | 0 |
| **LFE** | **DB** | 125 | 105 | 85 | 75 | 12 | 12 | 10 | 0 | 0 | 0 |
| **LFE** | **LFE** | 0 | 1 | 3 | 2 | 7 | 2 | 1 | 1 | 1 | 1 |
| **POP** | **LFE** | 0 | 3 | 1 | 0 | 0 | 0 | 0 | 0 | 0 | 0 |
|  | **POP** | 0 | 0 | 52 | 25 | 25 | 25 | 25 | 25 | 25 | 12 |
| **POP** | **DB** | 125 | 125 | 67 | 56 | 43 | 22 | 11 | 1 | 0 | 2 |
| **POP** | **PRR** | 0 | 1 | 1 | 2 | 7 | 0 | 1 | 0 | 0 | 0 |
| **var_0** | **var_1** | $\boldsymbol{\lambda}_{\boldsymbol{2}}$ **= 0.100** | $\boldsymbol{\lambda}_{\boldsymbol{2}}$ **= 1.075** | $\boldsymbol{\lambda}_{\boldsymbol{2}}$ **= 2.050** | $\boldsymbol{\lambda}_{\boldsymbol{2}}$**= 3.025** | $\boldsymbol{\lambda}_{\boldsymbol{2}}$**= 4.000** |  |  |  |  |  |
| **BD** | **PRR** | 0 | 0 | 1 | 1 | 0 |  |  |  |  |  |
|  | **LFE** | 8 | 3 | 4 | 8 | 7 |  |  |  |  |  |
| **BD** | **POP** | 1 | 1 | 0 | 1 | 1 |  |  |  |  |  |
| **BD** | **BD** | 121 | 121 | 112 | 23 | 1 |  |  |  |  |  |
| **PRR** | **BD** | 128 | 113 | 134 | 138 | 137 |  |  |  |  |  |
|  | **PRR** | 22 | 22 | 22 | 22 | 22 |  |  |  |  |  |
|  | **LFE** | 25 | 19 | 13 | 25 | 18 |  |  |  |  |  |
|  | **POP** | 51 | 47 | 52 | 50 | 50 |  |  |  |  |  |
| **LFE** | **POP** | 25 | 25 | 25 | 25 | 25 |  |  |  |  |  |
| **LFE** | **PRR** | 125 | 25 | 12 | 12 | 12 |  |  |  |  |  |
| **LFE** | **BD** | 128 | 113 | 134 | 138 | 137 |  |  |  |  |  |
| **LFE** | **LFE** | 111 | 100 | 100 | 23 | 32 |  |  |  |  |  |
| **POP** | **LFE** | 1 | 1 | 0 | 0 | 1 |  |  |  |  |  |
|  | **POP** | 16 | 20 | 19 | 17 | 17 |  |  |  |  |  |
| **POP** | **PRR** | 1 | 1 | 0 | 1 | 1 |  |  |  |  |  |
| **POP** | **BD** | 116 | 102 | 109 | 107 | 107 |  |  |  |  |  |
| **var_0** | **var_1** | $\boldsymbol{\lambda}_{\boldsymbol{3}}$ **= 0.100** | $\boldsymbol{\lambda}_{\boldsymbol{3}}$ **= 1.075** | $\boldsymbol{\lambda}_{\boldsymbol{3}}$ **= 2.050** | $\boldsymbol{\lambda}_{\boldsymbol{3}}$ **= 3.025** | $\boldsymbol{\lambda}_{\boldsymbol{3}}$ **= 4.000** |  |  |  |  |  |
| **BD** | **PRR** | 1 | 0 | 0 | 1 | 0 |  |  |  |  |  |
|  | **LFE** | 23 | 23 | 23 | 25 | 29 |  |  |  |  |  |
| **BD** | **BD** | 11 | 10 | 10 | 8 | 1 |  |  |  |  |  |
| **BD** | **POP** | 5 | 2 | 1 | 0 | 1 |  |  |  |  |  |
| **PRR** | **BD** | 81 | 60 | 92 | 87 | 79 |  |  |  |  |  |
|  | **PRR** | 25 | 25 | 25 | 25 | 25 |  |  |  |  |  |
|  | **LFE** | 13 | 23 | 24 | 17 | 21 |  |  |  |  |  |
|  | **POP** | 55 | 55 | 42 | 47 | 59 |  |  |  |  |  |
| **LFE** | **POP** | 25 | 25 | 25 | 25 | 25 |  |  |  |  |  |
| **LFE** | **BD** | 125 | 125 | 125 | 125 | 125 |  |  |  |  |  |
| **LFE** | **PRR** | 12 | 11 | 10 | 9 | 8 |  |  |  |  |  |
| **LFE** | **LFE** | 1 | 1 | 1 | 0 | 1 |  |  |  |  |  |
| **POP** | **LFE** | 1 | 1 | 0 | 1 | 0 |  |  |  |  |  |
|  | **POP** | 19 | 17 | 19 | 15 | 16 |  |  |  |  |  |
| **POP** | **PRR** | 1 | 1 | 0 | 1 | 1 |  |  |  |  |  |
| **POP** | **BD** | 89 | 79 | 91 | 95 | 96 |  |  |  |  |  |
| **var_0** | **var_1** | $\boldsymbol{\lambda}_{\boldsymbol{4}}$ **= 0.100** | $\boldsymbol{\lambda}_{\boldsymbol{4}}$**= 1.075** | $\boldsymbol{\lambda}_{\boldsymbol{4}}$ **= 2.050** | $\boldsymbol{\lambda}_{\boldsymbol{4}}$ **= 3.025** | $\boldsymbol{\lambda}_{\boldsymbol{4}}$ **= 4.000** |  |  |  |  |  |
| **BD** | **PRR** | 0 | 1 | 0 | 1 | 0 |  |  |  |  |  |
|  | **LFE** | 24 | 23 | 26 | 23 | 21 |  |  |  |  |  |
| **BD** | **BD** | 56 | 34 | 22 | 12 | 1 |  |  |  |  |  |
| **BD** | **POP** | 1 | 0 | 5 | 4 | 1 |  |  |  |  |  |
| **PRR** | **BD** | 180 | 188 | 185 | 186 | 184 |  |  |  |  |  |
|  | **PRR** | 25 | 25 | 25 | 25 | 25 |  |  |  |  |  |
|  | **LFE** | 12 | 17 | 18 | 19 | 22 |  |  |  |  |  |
|  | **POP** | 55 | 51 | 50 | 54 | 52 |  |  |  |  |  |
| **LFE** | **POP** | 12 | 15 | 12 | 15 | 1 |  |  |  |  |  |
| **LFE** | **PRR** | 1 | 1 | 0 | 0 | 0 |  |  |  |  |  |
| **LFE** | **BD** | 125 | 125 | 125 | 125 | 125 |  |  |  |  |  |
| **LFE** | **LFE** | 32 | 1 | 0 | 0 | 12 |  |  |  |  |  |
| **POP** | **LFE** | 0 | 1 | 0 | 2 | 1 |  |  |  |  |  |
|  | **POP** | 20 | 21 | 19 | 18 | 19 |  |  |  |  |  |
| **POP** | **PRR** | 2 | 1 | 9 | 8 | 9 |  |  |  |  |  |
| **POP** | **DB** | 122 | 121 | 117 | 118 | 116 |  |  |  |  |  |

**Table 1c**:  It shows the results of the model 3 (PRR case), identifying which hyperparameters are most influential in causing sign changes in the Impulse Response Functions (IRFs) and at which specific values these directional shifts are observed.
