# Supplementary material for: Wealth creation and disease burden: Evidence from Nigeria based on a Bayesian-VAR approach
Source: PLoS One. 2025 Nov 10;20(11):e0334709. doi: 10.1371/journal.pone.0334709 (PMC12599923; doi:10.1371/journal.pone.0334709)

**Supplementary materials**

**S2 file. Impulse response functions with the reverse ordering**

In this section we change the order of the variables relative to the the BVAR model in “**ORDER II**”, “**ORDER III**”, “**ORDER IV**” and show the new IRFs according to the new orders as follows.

Collectively, we obtained very similar results, allowing us to conclude that the ordering of the variables does not affect our analysis.

**ORDER II**

**Figure 1: BD, POP, LFE vs EB (model 1-EB model)**


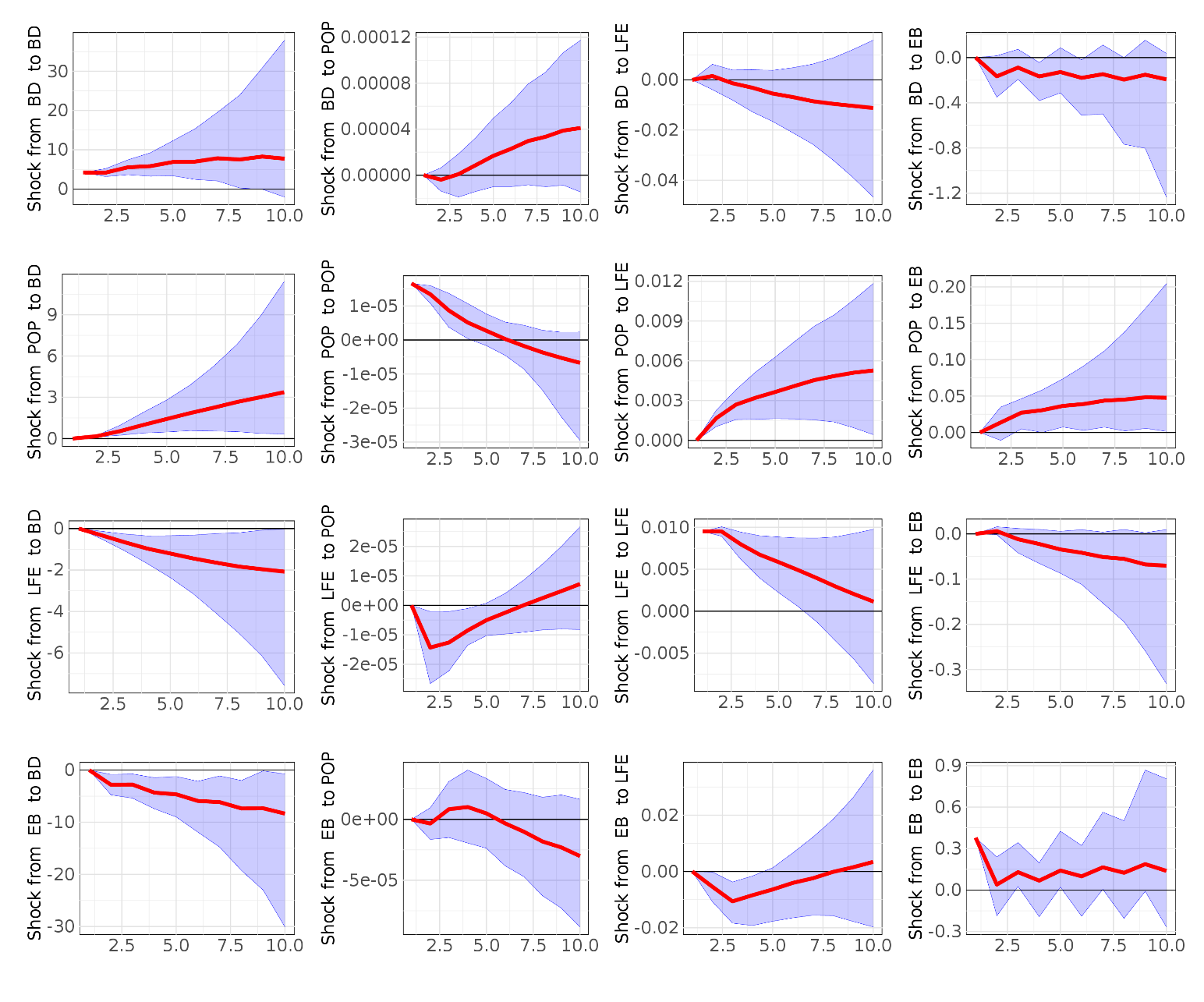


**Figure 2: BD, POP, LFE vs TIS (model 2-TIS model)**


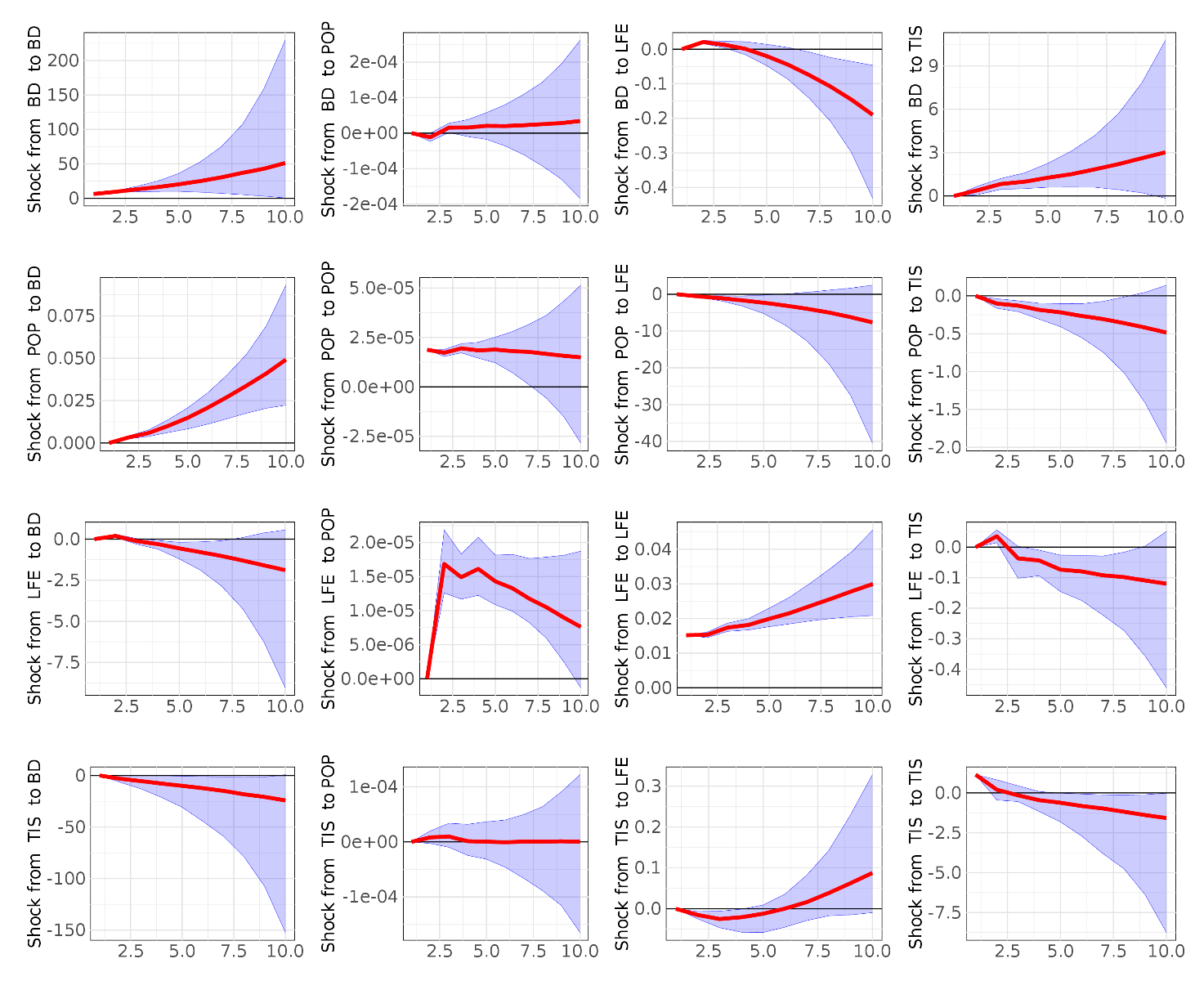


**Figure 3: BD, POP, LFE vs PRR (model 3-PRR model)**


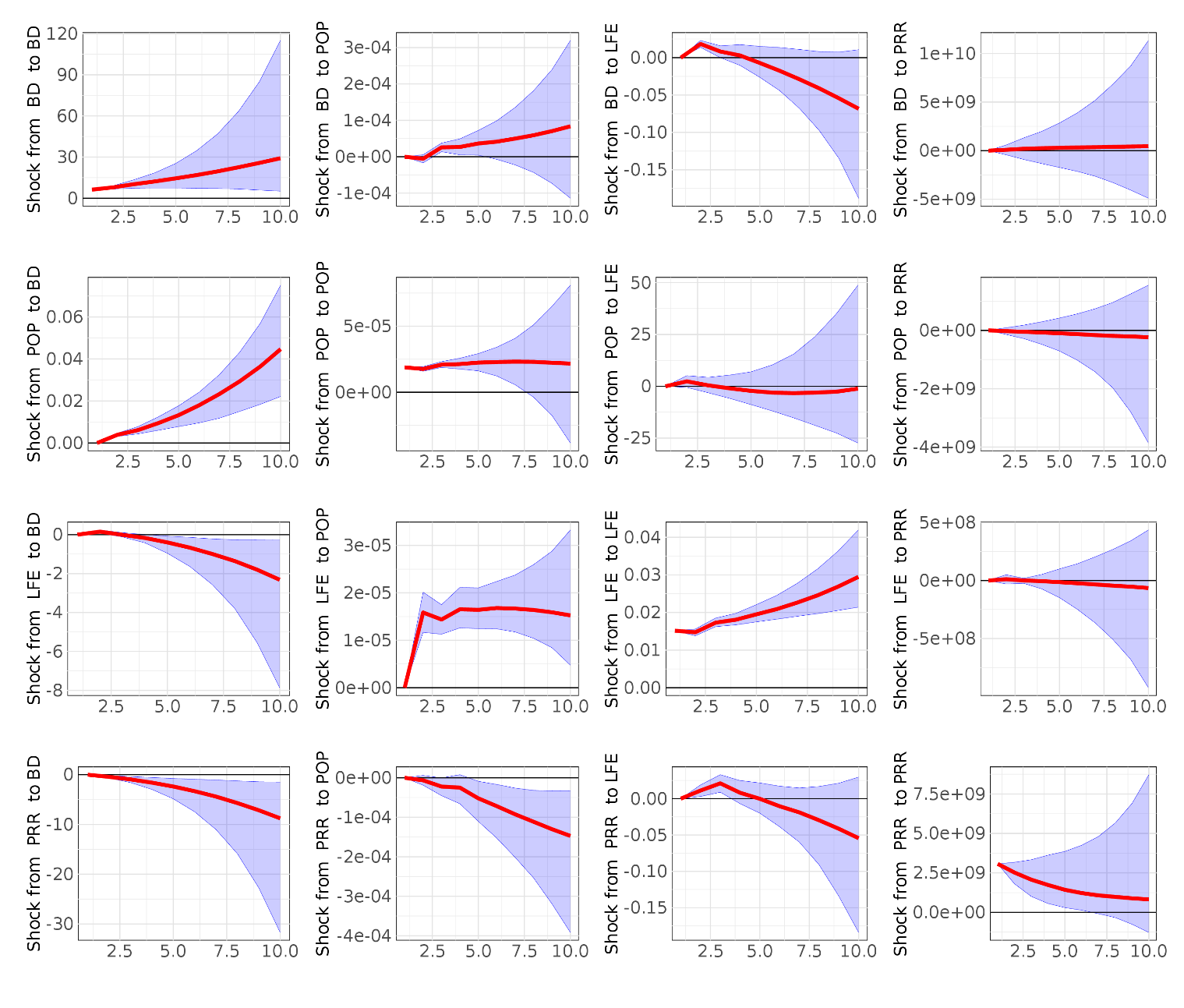


**ORDER III**

**Figure 4: EB, BD, LFE vs POP (model 1-EB model)**


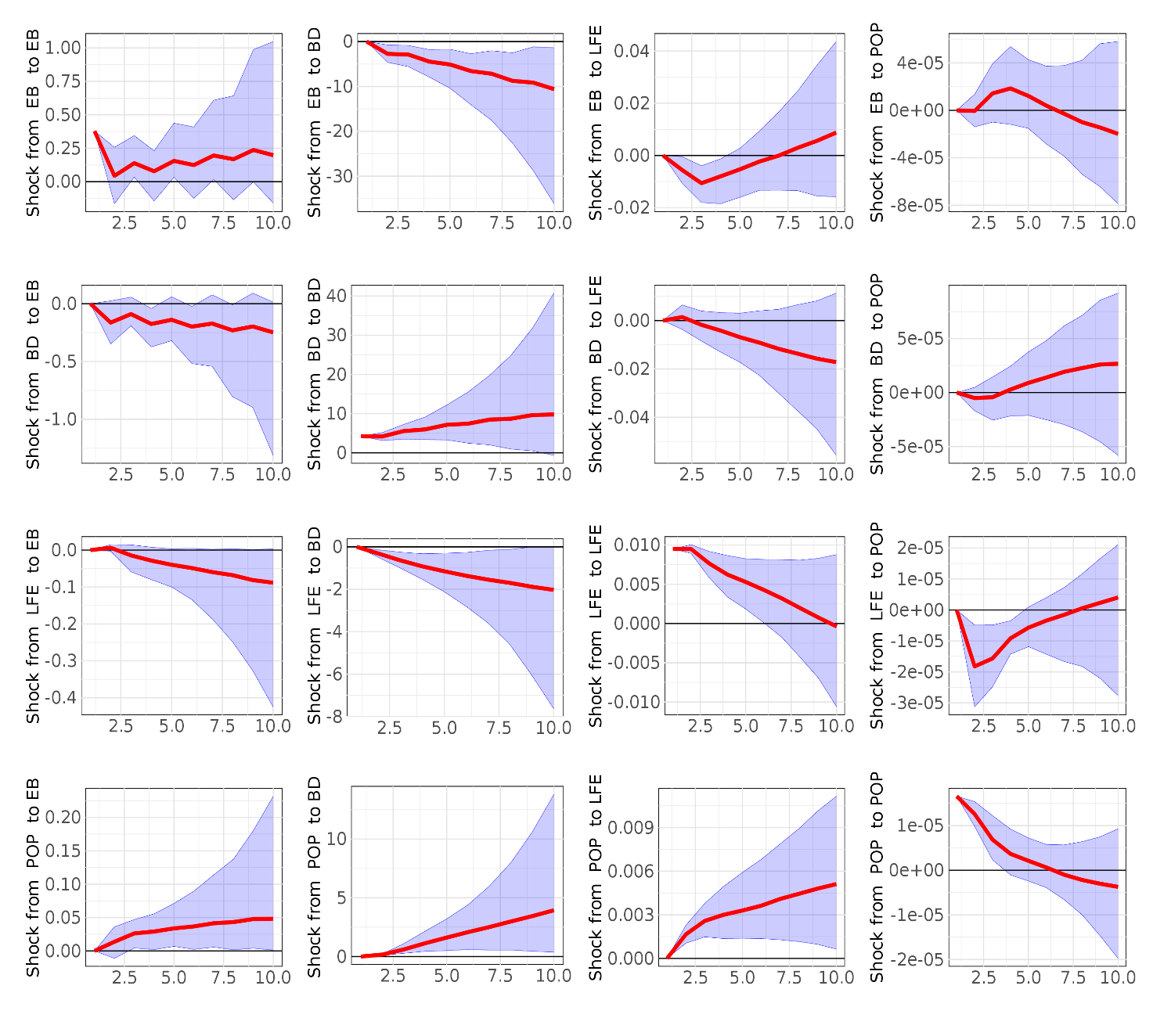


**Figure 5: TIS, BD, LFE vs POP (model 2-TIS model)**


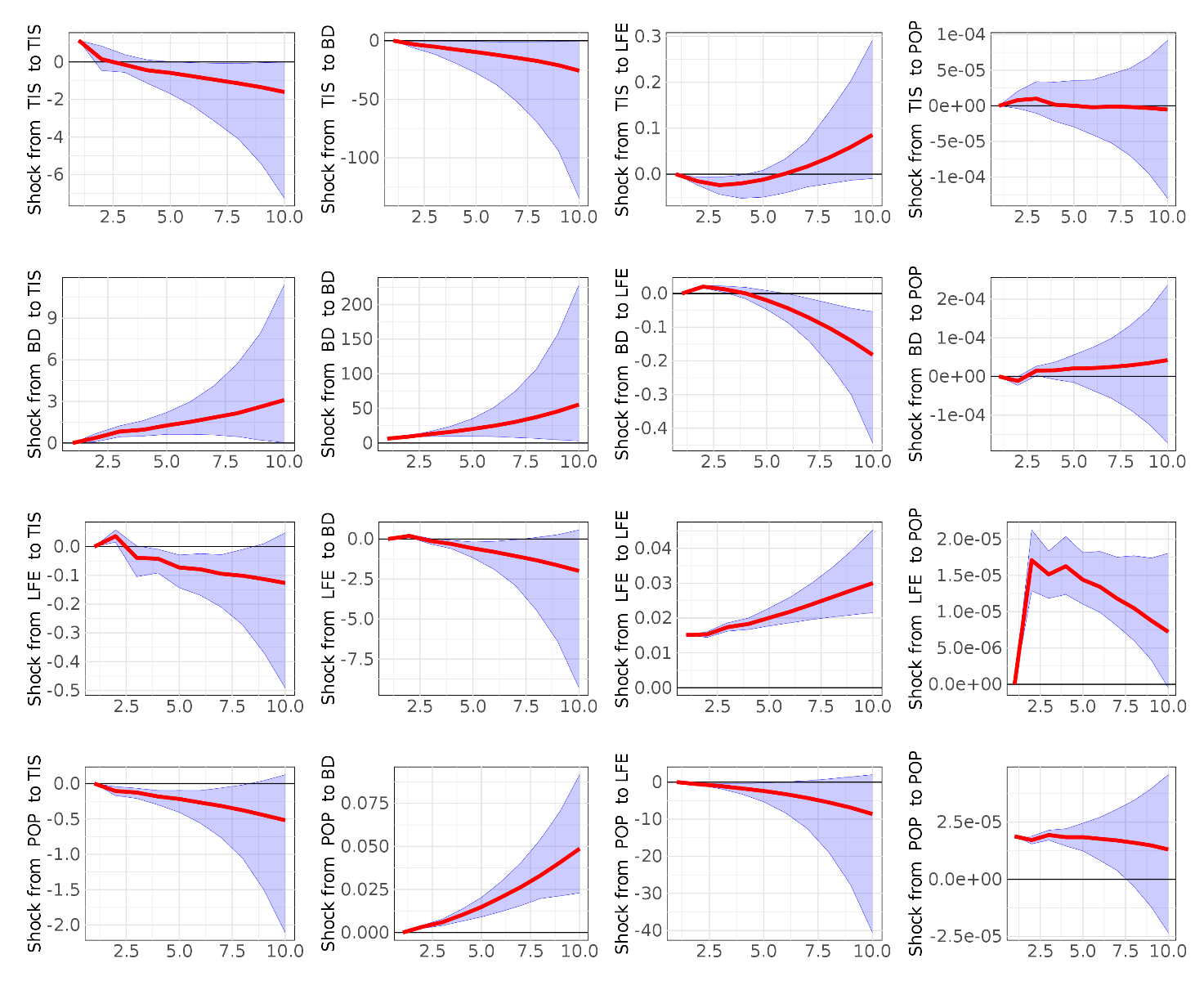


**Figure 6: PRR, BD, LFE vs POP (model 3-PRR model)**

**
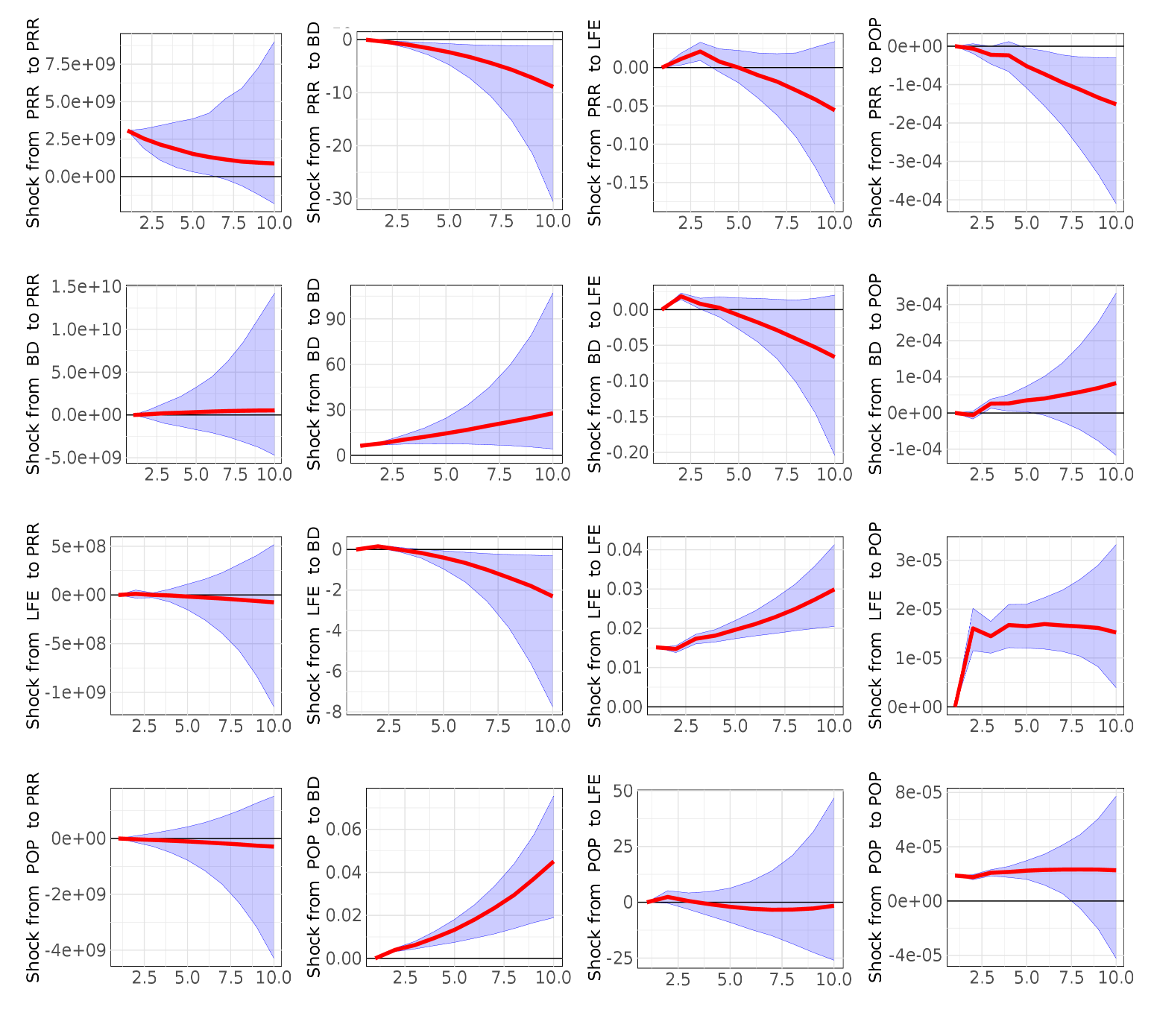
**

**ORDER IV**

**Figure 7:EB, POP, BD vs LFE (model 1-EB model)**


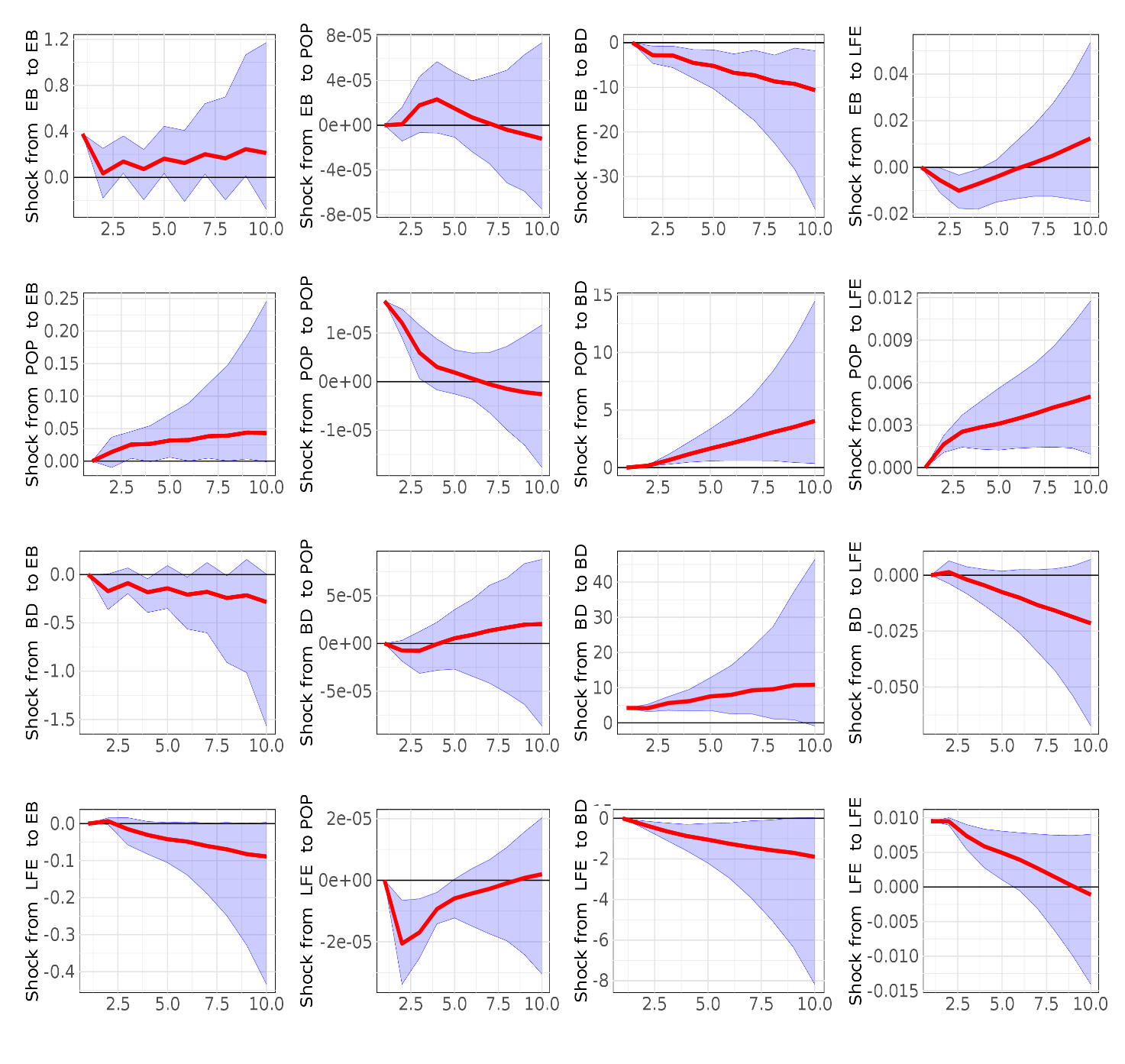


**Figure 8: TIS, POP, BD vs LFE (model 2-TIS model)**


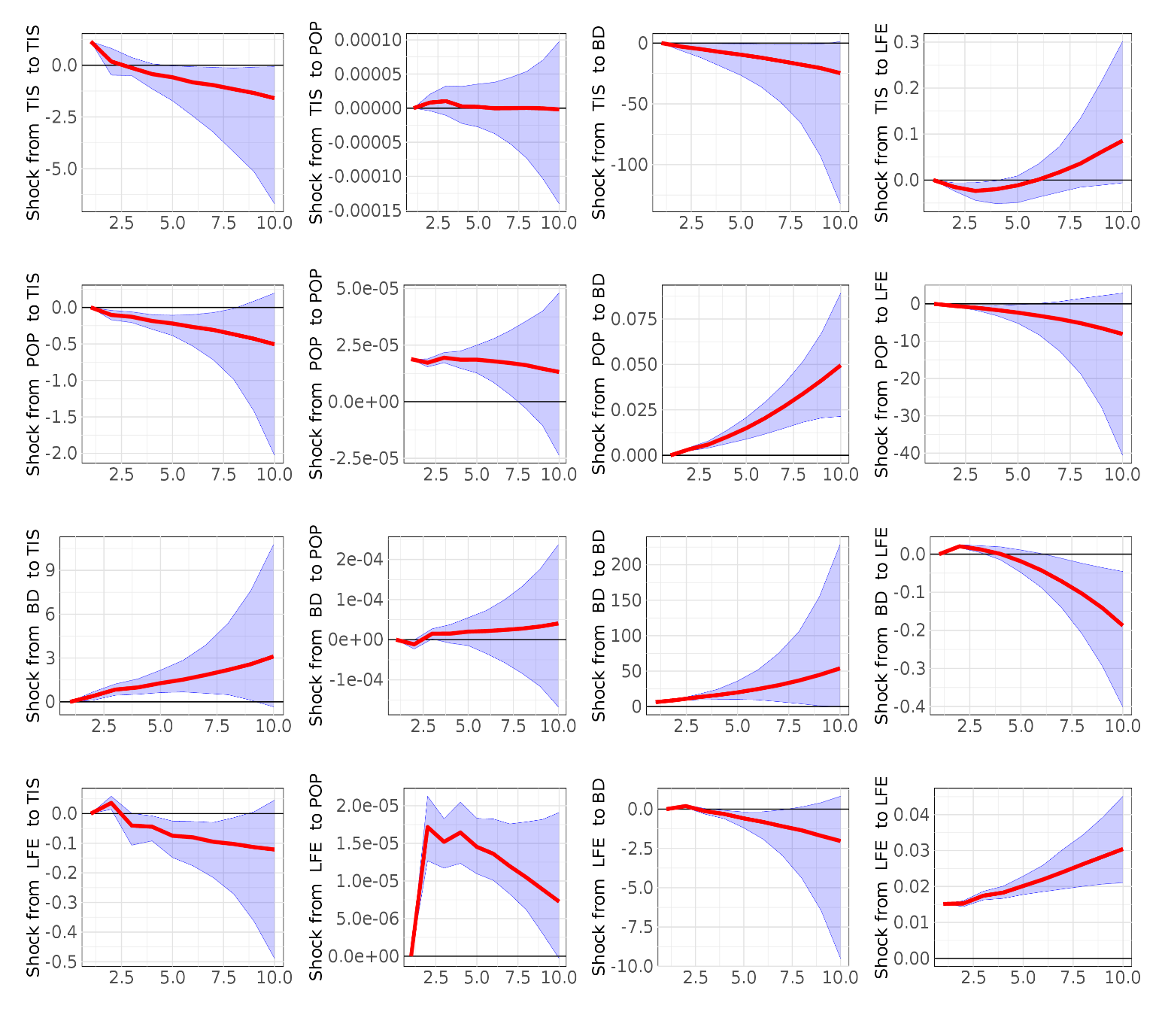


**Figure 9: PRR, POP, BD vs LFE (model 3-PRR model)**


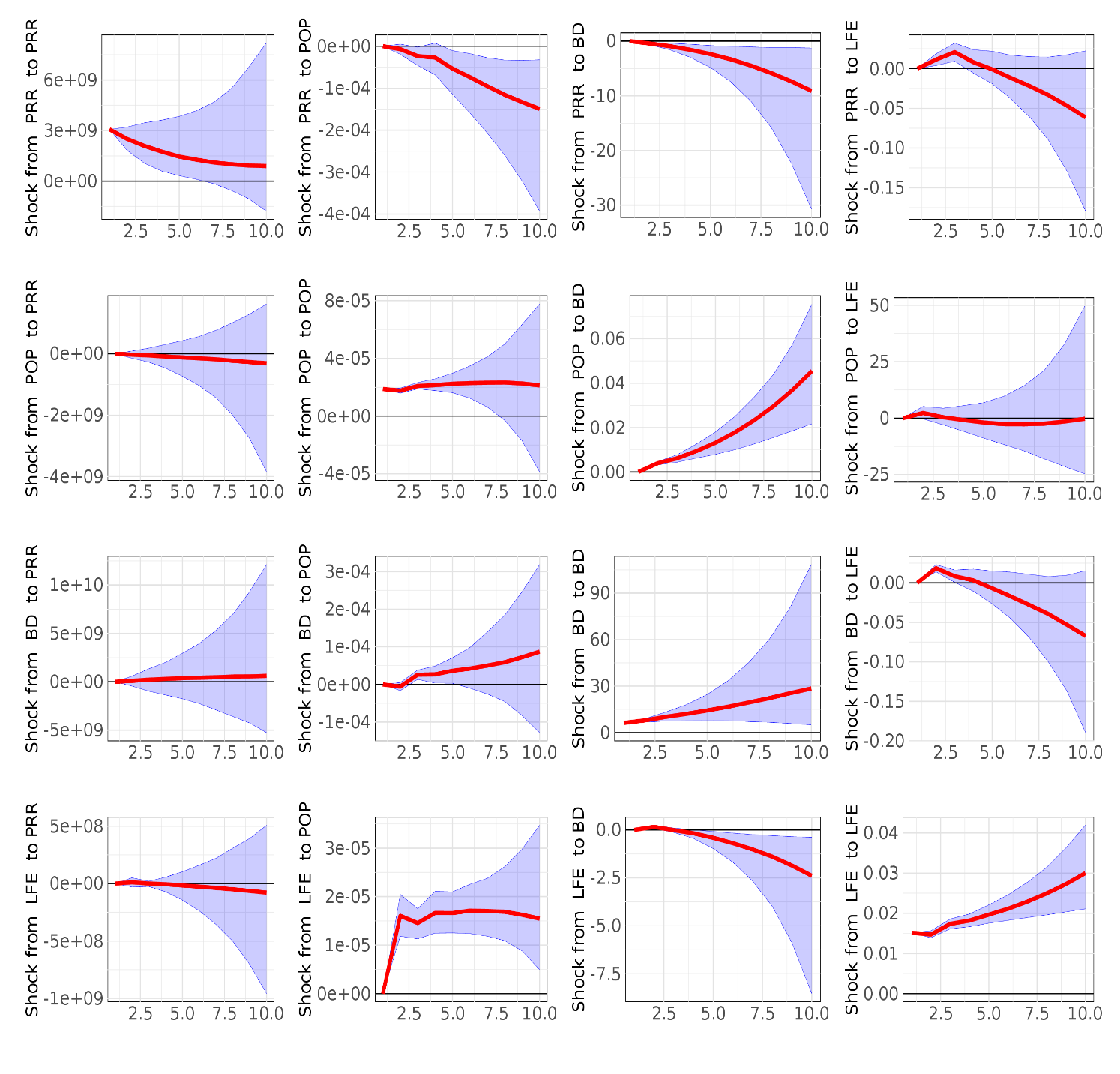

Supplement: S2 File — (DOCX) [file pone.0334709.s005.docx]
